# Effect of High-Pressure Processed Apples on Phenolic Metabolites, Short-Chain Fatty Acids, and Human Gut Microbiota Using a Dynamic In Vitro Colonic Fermentation System

**DOI:** 10.3390/metabo15120775

**Published:** 2025-11-29

**Authors:** Begoña de Ancos, Irene Fernández-Jalao, Claudia Balderas, Lucía Giménez, Concepción Sánchez-Moreno

**Affiliations:** 1 Department of Characterization, Quality and Safety, Institute of Food Science, Technology and Nutrition (ICTAN), Spanish National Research Council (CSIC), 28040 Madrid, Spain; lucia.gimenez@ictan.csic.es (L.G.); csanchezm@ictan.csic.es (C.S.-M.); 2 Faculty of Health Sciences, Universidad Francisco de Vitoria, Pozuelo de Alarcón, 28223 Madrid, Spain; irene.fernandezjalao@ufv.es; 3 Institute of Organic Chemistry (IQOG), Spanish National Research Council (CSIC), 28006 Madrid, Spain; claudia.balderas@iqog.csic.es

**Keywords:** high-pressure processing (HPP), apple, in vitro dynamic model of colonic fermentation, phenolic metabolites, gut microbiota, short-chain fatty acids (SCFAs)

## Abstract

**Background/Objectives**: Consuming apples regularly has positive effects on human health due to their anti-inflammatory and antioxidant properties, which have been associated with their phenolic composition. To enhance the bioactive properties of apple phenolic compounds, high-pressure processing (HPP) has been studied as a tool to improve their extraction during gastrointestinal digestion with the aim of increasing their bioaccessibility and the amount that reaches the colon unchanged, which can serve as substrates for bacterial fermentation. This study aimed to analyze the impact of an HPP-apple ingredient on the metabolism of human gut microbiota using an *in vitro* dynamic simulator of gastrointestinal digestion and colonic fermentation (GID-CF) that allowed us to study the three colon regions separately (ascending—AC; transverse—TC; and descending—DC). **Methods**: Apples were HPP-treated (400 MPa/5 min) and lyophilized to obtain an HPP-apple ingredient in powder form. A GID-CF was employed to study the continuous intake of the HPP-apple ingredient for 14 days at 37.5 g/day. **Results**: The HPP-apple ingredient produced a significant accumulation of phenolic metabolites mainly in the DC, with benefits on human health. The main phenolic metabolites formed were phloroglucinol, 4-hydroxyphenylacetic acid, 4-hydroxy-3-methoxyphenylacetic acid, 3-(3-hydroxyphenyl)-propionic acid, and 3-(4-hydroxyphenyl)-propionic acid. A PCA revealed a perfect separation of the three colon regions based on the phenolic precursors and metabolites. The microbiota-modulatory effects were attributed to the increase in *Bifidobacterium* spp. and *Lactobacillus* spp. populations and the butyric acid (SCFA) concentration. **Conclusions**: The results obtained highlight the health benefits and potential prebiotic-like effect of the HPP-apple ingredient on the gut microbiota.

## 1. Introduction

Apples (*Malus domestica* Borkh.) are one of the most consumed fruits that are available worldwide throughout the year. Their intake in the Western diet provides vital nutrients and health-promoting phytochemicals, mainly phenolic compounds and dietary fiber [1,2]. Around 70–75% of apples are consumed as fresh fruit, while the rest are processed into various products: apple juice or concentrate, apple cider, apple vinegar, freeze/dried apples, and apple sauce and jam [2].

Considerable epidemiological and scientific evidence indicates that frequent apple intake has positive effects on humans [3,4,5,6,7]. The health benefits of eating apples are strongly linked to their phenolic compounds [8,9,10].

The main phenolic compounds in apples include hydroxybenzoic acids (gallic acid and protocatechuic acid), hydroxycinnamic acids (mainly chlorogenic acid), flavanol monomers [(+)-catechin and (−)-epicatechin] and flavanol oligomers (procyanidins B1 and B2), flavonols (quercetin glucosides), and an appreciable amount of dihydrochalcones (phloretin-2′-glucoside and phloretin-2′-xyloglucoside) [1,11]. These phenolic compounds have a wide variety of beneficial effects on health, such as antioxidant, anti-inflammatory, cardioprotective, hepatoprotective, anticarcinogenic, antidiabetic, anti-obesity, and neuroprotective properties [8,9]. Apples are the main dietary source of chlorogenic acid and quercetin because they are frequently consumed worldwide and throughout the year. In apples, quercetin is glycosylated with several sugars, including galactose, glucose, rhamnose, xylose, and arabinose [11]. Their molecular configurations enable chlorogenic acid and quercetin to exhibit significant antioxidant properties, effectively neutralizing reactive oxygen species and free radicals, even inhibiting prooxidant enzymes (lipoxygenase, xanthine oxidase, NADPH oxidase). Moreover, quercetin and chlorogenic acid have cardioprotective effects related to their anti-inflammatory activity, platelet anti-aggregating effect, and vasodilator effect, as well as their ability to inhibit LDL oxidation and reduce adhesion molecules [1,12,13].

The health benefits of apple consumption depend on the types and amounts of phenolic compounds, which are influenced by cultivar, growing conditions, post-harvest handling, and processing [14].

Before apple phenolic compounds can exert their biological functions, they first undergo a two-stage process: release from the food matrix during digestion (bioaccessibility) followed by transport to systemic circulation and target organs (bioavailability). The absorption of phenolic compounds is influenced by their physicochemical properties (molecular size, structure, degree of polymerization/glycosylation, solubility, and conjugation with other molecules) [15], food matrix characteristics, and interferences with other dietary compounds (carbohydrates, lipids, proteins, fiber, etc.) [16]. Food processing can also induce chemical or physical changes in the food matrix that could affect the bioaccessibility of phenolic compounds [15,17].

Regardless of the strategy used to improve the bioaccessibility of phenolic compounds, only 5–10% are absorbed in the small intestine. A larger percentage (90–95%) reaches the large intestine, where the native microbiota converts them into low-molecular-weight catabolites. Thus, dietary phenolic compounds and other low-bioaccessibility compounds, such as dietary fiber (mainly pectin in apples), enter unchanged and simultaneously into the colon where they are biotransformed by the gut microbiota into low-molecular-weight phenolic compounds and short-chain fatty acids (SCFAs), respectively [18,19]. These phenolic compound metabolites formed in the gut are mainly responsible for the health benefits associated with the ingestion of foods rich in phenolic compounds. Furthermore, these compounds can potentially exert anti-inflammatory, antioxidant, antiproliferative, and antidiabetic effects in the colon [18,19,20,21].

In this sense, the phenolic compounds that reach the colon, in addition to acting as antimicrobial agents against pathogens, can act as prebiotic molecules that modulate the intestinal microbiota and prevent microbial imbalances (dysbiosis) linked to various gastrointestinal pathologies. To achieve this, phenolic compounds could increase the abundance of probiotics, like *Lactobacillus* spp., *Bifidobacterium* spp. and *Eubacterium* rectale-C. coccoides, and decrease *Clostridium* spp. and *Firmicutes* (among others), which are related to irritable bowel syndrome (IBS) and metabolic disorders, respectively [22]. Thus, a healthy gut microbiota plays a fundamental role in human health, as it protects against the entry of pathogens, maintains the structural integrity of the intestinal mucosal barrier, modulates metabolic and immune processes, participates in the communication pathways between the gut microbiota and the brain, and is closely related to protection against the development of degenerative disease, such as Alzheimer’s disease and age-related diseases [18,23,24,25].

The three main short-chain fatty acids (SCFAs) produced by the gut microbiota are acetate (C2), propionate (C3), and butyrate (C4), which are found in a molar ratio of approximately 60:20:20. However, the short-chain fatty acids (SCFAs) derived from proteins and peptides are branched-chain fatty acids (BCFAs), including 2-methylbutyrate, isobutyrate, and iso-valerate. After absorption in the colon, SCFAs serve as an energy source for gluconeogenesis and lipid synthesis while also regulating host immunity, inflammation, and oxidative stress [26,27].

The SCFAs produced by gut bacteria exert various regulatory effects on the human body, such as impacting gut ecosystem composition, intestinal barrier integrity, appetite-regulating hormone production, energy homeostasis, immune cell function, and circadian clock entrainment. Conversely, alterations in short-chain fatty acids (SCFAs) are associated with a wide range of disorders, such as diabetes, obesity, non-alcoholic fatty liver disease, hypertension, ulcerative colitis, Parkinson’s disease, and colorectal cancer [26,27]. Phenolic compounds can affect SCFA production as well. Thus, increased SCFA production has been linked to the colonic fermentation of phenolic compounds. Furthermore, phenolic compounds increase SCFA production by modifying the populations of SCFA-producing bacteria [26,27,28].

Nowadays, different non-thermal technologies, such as pulsed electric fields (PEF), high-pressure processing (HPP), ultrasounds (US), and high-pressure homogenization (HPH) have been proposed as alternatives to conventional thermal processing for controlling microbial growth and enzyme activity while preserving the health-related and sensory properties of plant-based foods [29,30,31]. PEF, US, HPP, and HPH can induce permeability changes in cell membranes through microstructural changes in solid matrices and particle size reduction in liquid matrices. These changes facilitate the release of health-related compounds including phenolic compounds and carotenoids, thereby improving their bioaccessibility. Thus, these technologies are being explored as tools to enhance the nutritional quality of plant-based products by increasing the bioaccessibility of bioactive compounds, such as phenolics, carotenoids, and vitamins, while minimizing the degradation of heat-sensitive nutrients [32,33,34]. Their effectiveness, however, depends on various factors, including the plant matrix and the compounds involved. Among these technologies, PEF and HPP are the most widely studied for improving the bioaccessibility of bioactive compounds, although they act through different mechanisms. PEF is particularly effective inducing pore formation in cell membranes, while HPP modifies cell structure for promoting the release of bioactive compounds [30,33]. Nowadays, optimizing the use of non-thermal technologies to improve the bioaccessibility of bioactive compounds in plant-based products requires the right combination of technologies and a comprehensive understanding of the effect of the food matrix [32,34].

The present study aimed to evaluate the impact of a 14-day continuous intake of an HPP-treated apple (400 MPa/5 min/35 °C) on the composition and functionality of the human gut microbiota using an in vitro gastrointestinal model that reproduces the physical and chemical conditions of both digestion and colonic fermentation simultaneously. Gut metabolic activity was assessed by analyzing the disappearance of phenolic compounds present in the HPP-apple ingredient and the formation of its metabolites, short-chain fatty acids, and changes in the microbial population in the three different regions of the colon (ascending, transverse, and descending colon).

## 2. Materials and Methods

### 2.1. Reagents

HPLC/MS-grade methanol was acquired from J.T. Baker (Deventer, The Netherlands). The other solvents were HPLC-grade and obtained from Lab-Scan (Dublin, Ireland). The commercial standards and chemical products used in the different analyses were supplied by Sigma Aldrich (St. Louis, MO, USA).

### 2.2. Raw Material

Apples (*Malus domestica* Borkh., ‘Golden Delicious’) from Aragon (Interlazaro S.L.U., Calatayud, Zaragoza, Spain) were acquired from a supermarket in Madrid (water content, 84.25 ± 3%; soluble solids (° Brix), 12.40 ± 0.35; pH, 3.85; fruit weight, 181.52 ± 8.23 g). The apples were stored at 4 °C until processing. The apples were washed, cut into 2 cm cubes with peel and no core, packaged at 200 g in low-permeability plastic bags (BB4L, Cryovac, Barcelona, Spain), and lightly vacuum-sealed. Two batches of apple slices were separated. One batch corresponded to the unprocessed product and the other to the product processed by HPP.

### 2.3. High-Pressure Processing (HPP) and Lyophilization

The two bags of packaged apples were placed into a 1925 mL vessel unit filled with water using Stansted HPP equipment (High-Pressure Iso-Lab System, Model FPG7100:9/2C, Stansted Fluid Power, LTD., Essex, UK). The samples were processed at 400 MPa for 5 min at 35 °C (30° initial temperature), with a pressure increase time of 3 MPa/s, according to previous results of the research group [34]. The process involved continuous, computerized control and monitoring of pressure, time, and temperature, with automatic data recording.

The HPP-treated and untreated apple samples were immediately frozen with liquid nitrogen and stored at −80 °C until lyophilized (100 mTorr, −90 °C) (model Lyoalfa, Telstar S.A., Barcelona, Spain). Then, the samples were pulverized in an ultracentrifugal grinder ZM 200 (Retsch GmbH, Haan, Germany) to obtain a fine powder (final particle size ≤ 0.5 mm) and stored at −20 °C until analyzed. HPP-treated apple was labeled as HPP-apple ingredient.

### 2.4. Characterization of the HPP-Apple Ingredient

The physicochemical, chemical, and biochemical characteristics of the untreated and HPP-treated apple samples at 400 MPa/5 min/35 °C (HPP-apple ingredient) are shown in Table S1. Titratable acidity, pH, total vitamin C, ascorbic acid, total pectin content, degree of esterification (%) of pectins, and soluble, insoluble, and total fiber, in addition to polyphenoloxidase (PPO), peroxidase (PPO) activity, and soluble proteins, were determined according to Fernández-Jalao et al. (2019) [34]. The sugar and organic acid contents were analyzed according to Colina-Coca et al. (2014) [35].

### 2.5. In Vitro Gastrointestinal Dynamic Digestion and Colonic Fermentation (GID-CF) of the HPP-Apple Ingredient

The HPP-apple ingredient (37.5 g dw) was submitted to a simulated gastrointestinal digestion and colonic fermentation (GID-CF) in a dynamic model known as ColomSim. This equipment better simulated the real conditions of human gastrointestinal digestion than the static systems. The GID-CF system is a multi-compartmental, dynamic, and computer-controlled system developed at AINIA Technologic Center (Valencia, Spain). The conditions of the GID-CF process used were determined according to the INFOGEST method [36]. The GID-CF system and conditions were previously described in Fernandez-Jalao (2020) [37] and in Fernandez-Jalao et al. (2021) [38].

The maximum amount of the HPP-apple ingredient (37.5 g dw) supported by the system was used. Considering the average fresh weight of the apples (181.52 g), the edible percentage (80%), and the water content (84.25%), the amount of the HPP-apple ingredient employed in GID-CF corresponds to the daily intake of 1.3 ‘Golden Delicious’ apples.

**Figure 1:**
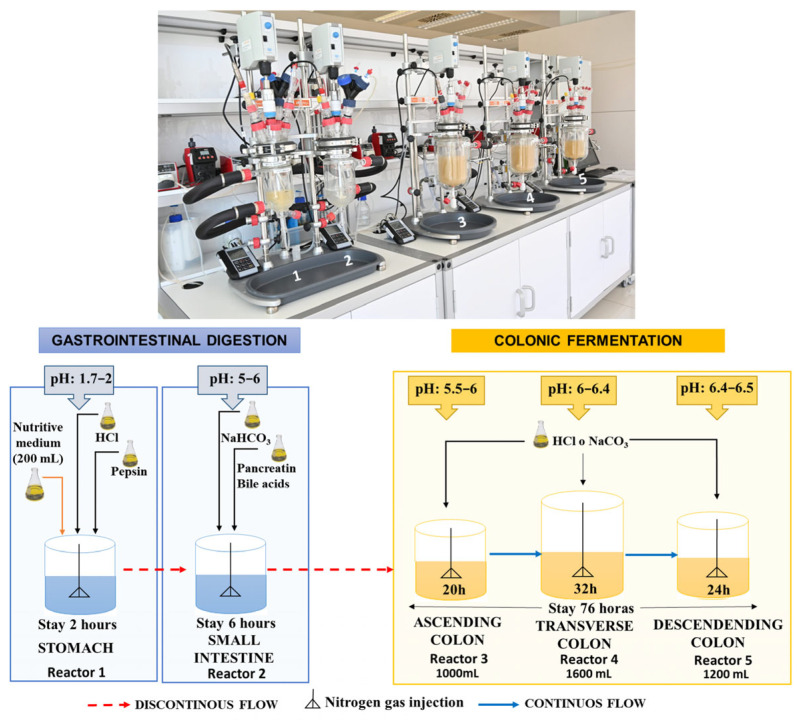
Dynamic in vitro gastrointestinal digestion and colonic fermentation (GID-CF) system (ColomSim, AINIA, Valencia, Spain) [37,38].

The GID-CF system consists of five reactors designed to model the stomach, small intestine, and ascending (AC), transverse (TC), and descending (DC) colonic processes (Figure 1). All the vessels were protected from light. The vessels simulating the stomach and small intestine were connected using peristaltic pumps in a semi-continuous manner. The three vessels simulating the colon (AC, TC, and DC) were also connected with peristaltic pumps, but in a continuous manner (Figure 1). All five vessels were agitated at 150 rpm, with all other process parameters, including pH, residence time, temperature (37 °C), and volume, precisely managed by a computer system. A continuous flow of gaseous N_2_ was maintained for 15 min, twice a day, in all five vessels to ensure anaerobic conditions.

Before colon fermentation, 37.5 g of the HPP-apple ingredient was submitted to a dynamic in vitro gastrointestinal digestion process (GID), according to Fernández-Jalao et al. (2021) [38] and Fernández-Jalao et al. (2017) [39]. First, the sample was mixed with 100 mL of artificial saliva (0.02 mol/L KCl, 0.005 mol/L KH_2_PO_4_, 0.02 mol/L NaHCO_3_, 0.2 mmol/L MgCl_2_∙6H_2_O, 0.02 mmol/L CaCl_2_) containing 75 units/mL of α-amylase to simulate the buccal phase (pH = 6). The mixture was increased to a final volume of 300 mL with Milli-Q water, homogenized for ten min, and bubbled with N_2_ gas before being introduced into the vessel to simulate the stomach. The gastric phase began by adding 60 mL of a simulated gastric electrolyte solution (0.009 mol/L KCl, 0.001 KH_2_PO_4_, 0.03 mol/L NaHCO_3_, 0.06 mol/L NaCl, 0.15 mmol/L MgCl_2_∙6H_2_O, 0.004 mmol/L CaCl_2_) containing pepsin (2000 U/mL). The small intestinal digestion phase was simulated by adding 240 mL of an electrolyte solution (0.008 mol/L KCl, 0.001 KH_2_PO_4_, 0.11 mol/L NaHCO_3_, 0.05 mol/L NaCl, 0.4 mmol/L MgCl_2_∙6H_2_O, 0.03 mmol/L CaCl_2_) containing pancreatin (800 U/L) and fresh bile (0.16 mmol/L). The pH in the stomach (pH 1.7–2) and small intestine (pH 5–6) was adjusted with 1M hydrochloric acid or sodium bicarbonate, respectively. The digested product resulting from gastrointestinal digestion (2 h + 6 h) was incorporated into the ascending colon three times a day (Figure 1).

A fermentation study was carried out using fecal samples from five healthy volunteers, three women and two men, between 35 and 50 years old, who signed an informed consent form. To be included, the volunteers had to be non-smokers, with no food allergies and no history of gastrointestinal or metabolic disorders, who followed a varied and balanced diet and did not take any vitamins or supplements. In addition, the volunteers did not take antibiotics in the three months before the study and followed a probiotic-free diet for 2 days before collecting the fecal sample. The fermentation process began on the same day the samples were collected. The study was carried out according to the Declaration of Helsinki, and the protocol was approved by the Ethics Committee of the AINIA Technology Centre (AINIA procedure n° 1180/15).

Before inoculation with human fecal samples (20%, *w*/*v*), the three colon vessels (AC, TC, and DC) were filled with the culture medium required for microbial inoculum growth. Also, the pH was set at 5.5–6 for the AC, 6–6.4 for the TC, and 6.4–6.8 for the DC. The three colon vessels were flushed with N_2_ for 15 min daily to ensure anaerobic conditions [37,38].

The experiment was conducted in two sequential parts: an initial stabilization period (basal) followed by the intake period. The 12-day stabilization period (basal) was necessary to allow the intestinal microbiota to adapt to the nutritional and physicochemical conditions of the vessels that simulated the three colon regions (AC, TC, and DC). To achieve this, the system was fed 200 mL of culture medium three times a day, administered from the stomach (Figure 1). The intake period consisted of feeding the system with 37.5 g of the HPP-apple ingredient with 200 mL of nutritive medium for 14 days. The culture medium alone was added to the system two more times per day. The volume and transit time of each compartment of the GID-CF simulating the in vivo residence time were 260 mL and 2 h in the stomach, 460 mL and 3 h in the small intestine, 1000 mL and 20 h in the AC, 1600 mL and 32 h in the TC, and 1200 mL and 24 h in the DC (Figure 1).

To study the metabolic activity of intestinal microbiota (phenolic metabolites and short-chain fatty acid analysis), a daily 50 g sample was collected from each of the three colon reactors (AC, TC, and DC) during the 14 days of the intake period. All these samples were quickly aliquoted and stored at −20 °C in darkness until they were transported from AINIA to ICTAN under controlled freezing conditions. Immediately upon arrival, the samples were stored at −80 °C for one week and then lyophilized (Lyophilizator model Lyoalfa, Telstar S.A, Barcelona, Spain). Following freeze-drying, the samples were manually ground using a home-use grinder and maintained at −20 °C until subsequent analysis.

To evaluate colonic microbiota modulation, a daily 5 g sample was collected from each of the three colon vessels (AC, TC, and DC) in aseptic conditions at specific time points during the stabilization period (0, 6, 8, 10, and 12 days) and during the intake period (1, 2, 6, 8, 10, and 14 days). These samples were immediately stored at 4 °C and analyzed within two hours at the AINIA microbiology laboratory. The whole GID-CF experiment was carried out twice.

### 2.6. HPLC-ESI-QTOF-MS/MS Analysis of Phenolic Compounds and Metabolites

#### 2.6.1. Extraction of the HPP-Apple Ingredient Before Digestion

Phenolic compounds were extracted according to a previously described procedure [38,39]. The HPP-apple ingredient (1 g) was homogenized (homogenizer model ES-270, Omni International Inc., Gainesville, Waltham, MA, USA) with 12.5 mL of a methanol and water mixture (80:20, *v*/*v*) at 8000 rpm for 4.5 min. The mixtures were centrifuged (8000× *g*, 4 °C, 15 min) (Thermo Scientific Sorvall, mod. Evolution RC, Thermo Fischer Scientific Inc., Waltham, MA, USA), and the pellet was re-extracted under the same conditions. The two supernatants were combined, evaporated at 40 °C using a vacuum evaporator, reconstituted with 10 mL of methanol, and stored at −20 °C until HPLC-ESI-QTOF-MS/MS analysis.

#### 2.6.2. Colonic Fermentation Slurry Extraction

Phenolic compounds and phenolic metabolites from the colonic fermentation dried slurry of the AC, TC, and DC were extracted according to Fernández-Jalo (2020) [37] and Fernández-Jalao et al. (2021) [38]. Samples of the lyophilized samples (25 mg) were mixed with methanol/acidified water (0.1% formic acid) (80:20 *v*/*v*) (1.5 mL), vortexed for 1 min, sonicated for 5 min, and centrifuged at 11,400× *g* for 15 min at 4 °C (Thermo Scientific Sorvall, mod. Evolution RC, Thermo Fisher Scientific Inc., Waltham, MA, USA). The supernatants were collected and re-centrifuged twice. The supernatant (1 mL) was diluted with 2 mL of ultrapure water [or 2 mL of methanol/water, 80:20 (*v*/*v*) for detection and quantification of quercetin aglycone]. The extractions were performed in triplicate (*n* = 3).

In addition, one quality control (QC) (mixture of all extracts) and one pool sample per colon region (a mixture of extracts from the AC, TC, and DC) were prepared. All extracts were filtered through a 0.22 µm filter before HPLC-ESI-QTOF-MS/MS analysis.

#### 2.6.3. HPLC-ESI-QTOF-MS/MS Analysis of Phenolic Compounds and Metabolites

The separation, identification, and quantification of phenolic compounds and their metabolites was achieved according to Fernández-Jalao (2020) [37] and Fernández-Jalao et al. (2021) [38] using a high-performance liquid chromatography (HPLC) system with a diode array detector (DAD) coupled to an Agilent 6530 Accurate-Mass Quadrupole Time-of-Flight (Q-TOF) mass spectrometer (MS) equipped with an Agilent Jet Stream dual electrospray ionization (ESI) source (Agilent Technologies Inc., Santa Clara, CA, USA). The mobile phase consisted of a gradient of 0.1% formic acid in Milli-Q water (A) and 0.1% formic acid in acetonitrile (B) applied as follows: 0 min, 95% A; 15 min, 80% A; 25 min, 50% A; 30 min, 20% A; and 40 min, 95% A. The flow rate was fixed at 0.4 mL/min, and the injection volume was 10 µL. Runs were monitored at 360 nm, 320 nm, and 280 nm.

To identify the phenolic compound precursors and their possible metabolites, total ion spectra were acquired over the 100–1000 *m*/*z* range in negative mode. Nitrogen was used as the drying, collision, and nebulizing gases. The drying gas temperature and flow rate were set at 225 °C and 10 L/min, respectively, while the sheath gas temperature and flow rate were 300 °C and 10 L/min. The nebulizer gas pressure, skimmer voltage, octopole RF, and fragmentor voltage were 45 psi, 65 V, 750 V, and 125 V, respectively. The capillary voltage was set at 3 kV, and the MS/MS collision energy was fixed at 20, 30, and 40 eV.

Data were acquired and analyzed using Agilent Masshunter Acquisition software (version B.07.00) (Agilent Technologies Inc, Santa Clara, CA, USA). Raw LC-MS data were cleaned of background noise and unrelated ions using the Molecular Feature Ex-traction (MFE) algorithm implemented in Mass Hunter Qualitative Analysis Software B.06.00 (Agilent Technologies, Inc., Santa Clara, CA, USA). The MFE generated a list of the ion characteristics for the phenolic compounds and their corresponding metabolite profiles; each compound was described by mass, retention time, and abundance. Post-acquisition processing was performed in Agilent Profinder B.08.00 (Agilent Technologies Inc., Santa Clara, CA, USA). Mass and retention time alignment, followed by a filter by frequency post-processing, was performed to retain only the features present in >70% of the samples within at least one treatment. The relative standard deviation (%RSD) of the compound concentrations in the QC (quality control) samples was calculated, and only compounds below the limit (30%) were considered for data treatment.

The identification was based on a comparison with the mass spectral data generated by external standards, the data from databases (MassBank, Pubchem, Phenol explorer, MoNa database), and a personal accurate mass database built using the information about mass data of the main phenolic compounds present in onion and their possible metabolites obtained from the literature.

Phenolic compound precursors and metabolites were quantified in MS1 mode using external calibration curves systematically injected every 10 runs of the sequence. Five-point calibration curves were constructed for each commercially available compound in the range of 5 to 0.005 µg/mL. When commercial standards were not available, quantification was carried out using structurally related standards. QC samples were prepared by mixing 10 µL of each sample extract. All analyses were performed in triplicate (*n* = 3).

### 2.7. Analysis of Short-Chain Fatty Acids (SCFAs)

Short-chain fatty acids (SCFAs) were extracted from the colonic fermentation slurry from the AC, TC, and DC reactors [37,40]. Samples of the colonic fermentation dried slurry (20 mg dw) were extracted and diluted with a 0.5% phosphoric acid solution to reach a final concentration of 0.005 g/mL. An aliquot (100 µL) of the diluted extract was mixed with 300 µL of 0.5% phosphoric acid and 100 µL of methyl valeric (788 µM) as an internal standard (prepared with 0.5% phosphoric acid). Then, the samples were extracted with 1000 µL of n-butanol. The standards used in this analysis were also extracted with n-butanol. Gas chromatography analyses were performed using the organic phase of n-butanol. SCFA determination was performed on an Agilent GC 6890 gas chromatograph with a flame ionization detector (FID) and an automatic injector (G2613A). A DB-WAXtr column (100% polyethylene glycol, 60 m, 0.325 × 0.25 mm) was used. Helium was used as carrier gas at a flow rate of 1.5 mL/min. The samples were injected in splitless mode (without flow division), with an injection volume of 1 µL and an injector temperature of 250 °C. The temperature gradient used was 2 min at 50 °C, a first ramp of 15 °C/min up to 150 °C, a second ramp of 5 °C/min up to 200 °C, and a last ramp of 15 °C/min up to 240 °C. The analysis was performed in 41.3 min. The FID temperature was set at 260 °C. The system was controlled with MSD-Chemstation software version E.02.00.493. To identify SCFAs, a standard solution (FAMQ-004 AccuStandard) containing acetic, propionic, isobutyric, butyric, isovaleric, valeric, caproic, and heptanoic acids was used. Quantification was performed using a standard WSFA-2 Mix (Supelco) containing acetic, propionic, butyric, and valeric acids.

### 2.8. Microbial Analysis 

The bacterial count was determined according to the procedures described in Fernández-Jalao (2020) [37] and Fernández-Jalao et al. (2021) [38]. While we are aware of faster, more sensitive, and specific advanced molecular methods, such as quantitative PCR and fluorescence in situ hybridization (FISH) with oligonucleotide probes targeting ribosomal RNA, we chose to use the traditional method for this study. Samples (5 g of fresh slurry, fw) from the three colon vessels (AC, TC, and DC) during the stabilization (at 0, 6, 8, and 12 days) and intake periods (at 0, 2, 6, 8, 10, and 14 days) were aseptically transferred to tubes containing Anaerobe Basal Broth (Oxoid, Thermo Scientific, Basingstoke, UK) and transported immediately to the microbiology laboratory of AINIA (Valencia, Spain), where they were analyzed within two hours. Specific groups of bacteria in the fermentation products were isolated and enumerated using selective and differential growth media and suitable incubation conditions [37,38]: VRBD Agar (Merck-Millipore, Burlington, MA, USA) for *Enterobacteriaceae*, VRBL Agar (Merck-Millipore) for total coliforms; TSC Agar (Oxoid) for *Clostridium* spp., Tos-propionate Agar (Merck-Millipore) for *Bifidobacterium* spp., Schaedler Anaerobe Agar (Oxoid) for total anaerobes, and MRS Agar (Oxoid) for *Lactobacillus* spp. All the plates were incubated at 37 °C in anaerobic conditions for 24 h (*Enterobacteriaceae*, *Clostridium* spp. and total coliforms), 48 h (*Bifidobacterium* spp. and *Lactobacillus* spp.), and 72 h (total anaerobes). *Lactobacillus* microorganisms on the MRS-agar plates were identified using MALDI-TOF mass spectrometry. Anaerobic bacteria and *Enterobacteriaceae* were analyzed as indicators of total colonic microbiota, total coliforms, and *Clostridium* spp. as a part of the regular microbiota composition. *Bifidobacterium* spp. and *Lactobacillus* spp. were associated with the beneficial microbiota of the colon. Data were expressed as the logarithm of colony-forming units per gram of fresh slurry (Log UFC/g fw). The analysis was performed in triplicate (*n* = 3).

### 2.9. Statistical Analysis and Metabolomic Analysis Using a Targeted Approach

The results are presented as mean values ± standard deviations (SDs) of at least three separate experiments (*n* = 3). To identify statistically significant differences (*p* ≤ 0.05) in the phenolic compounds and their metabolite contents, short-chain fatty acid production, and microbiota composition in the three colon regions on different fermentation days, one-way analysis of variance (ANOVA) was applied, followed by Tukey’s b post hoc test. Levene’s test was applied to verify the homogeneity of variances. All statistical analyses were performed using the IBM SPSS Statistics 30 Core System (SPSS Inc., an IBM Company, Armonk, NY, USA).

Multivariate analysis based on principal component analysis (PCA) was applied to evaluate the quality of data obtained with HPLC-ESI-QTOF-MS/MS using MetaboAnalyst 6.0 (XiaLab, McGill University, Montreal, QC, Canada) [41]. Pareto scaling was applied, and a logarithmic transformation was performed to approximate a normal distribution. To evaluate the quality of analytical data and to remove possible outliers, PCA was performed on the fecal and QC samples. The robustness of the analytical procedure was verified by clustering the QC samples, which reflects the system’s stability, analytical performance, and reproducibility of the sample treatment procedures.

To explore the relationships between phenolic compound profiles, gut bacterial composition, and short-chain fatty acid (SCFA) production across the different colon sections, Pearson correlation coefficients were calculated between each bacterial or SCFA class and the phenolic compound classes. The original datasets for bacteria, SCFAs, and phenolic compounds were first reshaped into long format, merged by SampleID, and then converted into comparable wide matrices. For phenolic compounds, relative abundances were aggregated by chemical class by summing all values corresponding to each category. Correlations were computed directly on the raw abundance values. Section-specific matrices (AC, TC, and DC) to evaluate local associations, and corresponding *p*-values were obtained; only statistically significant correlations (*p* < 0.05) were displayed in the heatmaps. Additionally, interaction networks were constructed by retaining only strong associations (|r| ≥ 0.8), enabling the identification of robust connections between phenolic classes and bacterial or SCFA profiles, and highlighting potential metabolic routes and fermentation patterns characteristic of each colon region.

## 3. Results and Discussion

### 3.1. Characterization of the Undigested HPP-Apple Ingredient

#### 3.1.1. Physicochemical, Chemical, and Biochemical Characteristics

High-pressure processing (HPP) at 400 MPa/5 min/35 °C of ‘Golden Delicious’ apples was selected according to the previous results of the research group [37,39]. The physicochemical, chemical, and biochemical characteristics of the untreated HPP-apple product used in the present study were similar to those previously described [34]. HPP at 400 MPa/5 min/35 °C did not modify either the physicochemical characteristics or the sugar and organic acid contents in the apples, as shown in Table S1. However, a significant decrease of 4.22% in the vitamin C content was observed. Also, the HPP-apple ingredient showed a 61% lower protein content than the untreated apple due to the protein denaturing effect produced by HPP. Additionally, peroxidase (POD) activity decreased by 45% after HPP treatment, although polyphenoloxidase (PPO) activity was maintained. The total fiber content was not modified by HPP, while insoluble fiber decreased in favor of soluble fiber, which increased by 24%. In addition, total pectin increased by 7.69% due to HPP. The HPP-apple ingredient stands out for its high pectin and maleic acid contents (Table S1).

#### 3.1.2. Phenolic Compound Content

The phenolic composition of the HPP-apple ingredient was similar to that previously described [34]. Thus, the HPP-apple ingredient (400 MPa/5 min/35 °C) was analyzed with HPLC-ESI-QTOF-MS/MS, and 27 phenolic compounds belonging to four different families (phenolic acids, flavonols, flavanols, and dihydrochalcones) were separated, tentatively identified, and quantified (Tables S2 and S3). The total phenolic content in the HPP-apple ingredient was 1426.75 µg/g of dry weight (dw) (Table S3). Phenolic acids, flavanols, flavonols, and dihydrochalcones accounted for 48.1%,32.4%, 13.2% and 6% of the total phenolic content, respectively (Table S3). In general, the concentration of the different phenolic compounds in the HPP-apple ingredient was significantly (*p* < 0.05) lower than in other similar products [32]. The composition of fruit phenolic compounds is very dependent on different factors, such as cultivar, ripening stage, agricultural practices, environmental factors, growing region, and post-harvest conditions, among others [14].

The main phenolic compounds in the undigested HPP-apple ingredient belonged to phenolic acids (686.13 ± 6.16 µg/g dw), where chlorogenic acid, neochlorogenic acid, and *p*-coumaroyl quinic acid contributed 53.6%, 28.9%, and 16.2% to the total phenolic acid content, respectively (Table S3). The second most abundant family of phenolic compounds in the nondigested HPP-apple ingredient was flavanols (462.58 ± 2.01 µg/g dw), where epicatechin and procyanidin B2 represented 50% and 27% of the total flavanols, respectively (Table S3). The flavonol concentration (187.81 ± 2.17 µg/g dw) was mainly composed of quercetin-3-*O*-rhamnoside and quercetin-3-*O*-galactoside, which represented 42.4% and 35.2% of the total flavonols, respectively. The last phenolic compound family in abundance was dihydrochalcones (86.93 ± 0.84 µg/g dw), where phloridzin (60%) and phloretin-2’-xyloglucoside (27.7%) were the main representative compounds of this group. In addition, 3-hydroxyphloretin-2’-xyloglucoside, 3-hydroxyphloretin-2’-glucoside, and phloretin-pentoxyl-hexoside were also identified [42] (Table S2). According to previous results [32], HPP (400 MPa/5 min/35 °C) significantly increased the concentration of certain phenolic compounds of ‘Golden Delicious’ apples, such as flavonols (between 20% and 30%). HPP is known to change the membrane permeability or disrupt cell walls, favoring the release of phenolic compounds from tissues and improving their extractability [43]. Other authors have also described this behavior in different apple-based products treated with HPP. For example, a significant increase in total phenolic compounds and epicatechin content (up to 85%) was observed in ‘Pink Lady’ juice after pretreating apple pulp with HPP (600 MPa/4–5 °C/3 min) [44]. Similarly, HPP (500 MPa/25 °C/3 min) increased the total phenolic content in ‘Fuji’ apple juice by 39% [45], and HPP at 450 MPa/25 °C/10 min significantly increased total phenolic compound, total flavonoid, and total flavonol contents and antioxidant capacity in apple juice [46].

### 3.2. Phenolic Compound Precursors and Metabolites in the Colon Fermentation Products of the HPP-Apple Ingredient: Metabolomic Analysis by a Targeted Approach

The phenolic compound precursors and phenolic metabolites found in the gut fermentation samples of the ascending colon (AC), transverse colon (TC), and descending colon (DC) during the 14-day treatment period simulating a 37.5 g daily ingestion of the HPP-apple ingredient were separated, identified, and quantified using HPLC-ESI-QTOF-MS/MS (Tables S2, S4 and S5).

#### 3.2.1. Evolution of Phenolic Compound Precursors During Colonic Fermentation

**Figure 2:**
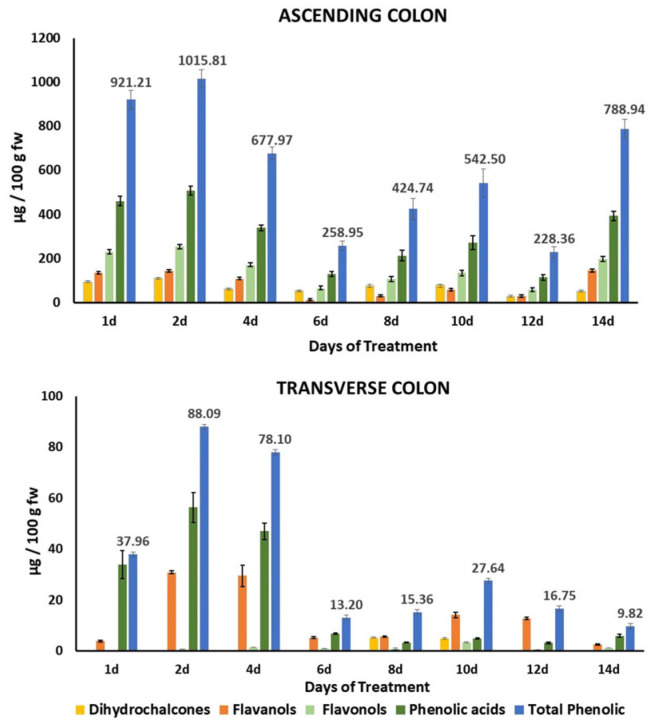
Evolution of phenolic precursors (total and grouped by families) in the gut fermentation slurry of the ascending (AC) and transverse (TC) colon. Data are expressed as mean ± standard deviation (*n* = 3); fw, fresh weight; d, day of treatment.

The bioaccessibility of the total phenolic compounds in HPP-apple was estimated at 10.89% [39], so most of these phenolic compounds reach the ascending colon (AC) and can serve as substrates for bacterial fermentation. Thus, continuously feeding the colon fermentation model with the gastrointestinal digested product from 37.5 g of the HPP-apple ingredient produced a significant accumulation of phenolic precursors, mainly in the AC, with a minor concentration in the TC and practically nothing in the DC (Figure 2, Table S4). In general, total phenolic precursors in the AC were significantly higher than in the TC on the same days of treatment: approximately 10 times between days 2 and 4, 22 times between days 6 and 10, and 79 times at treatment day 14 (Figure 2). The maximum amount of total phenolic precursors in the AC (1015.81 ± 40.33 µg/100 g of fresh weight (fw)) was counted on day 2 of the treatment, followed by a continuous decrease up to day 12 (228.36 ± 25.63 µg/100 g fw) (Figure 2).

Previous studies showed that when feeding the same dynamic fermentation model system with an HPP-onion ingredient instead of an HPP-apple ingredient, higher accumulation of phenolic precursors was observed, but only in the AC [38].

The main phenolic precursors found mainly up to day 14 of treatment were phenolic acids (mainly chlorogenic acid, cryptochlorogenic acid, and *p*-coumaroyl quinic acid), followed by flavonols (mainly Q-3-galactoside and Q-3-rhamnoside), flavanols (mainly epicatechin and procyanidin B2), and, finally, dihydrochalcones (mainly phloretin-2’-xyloglucoside and phloridzin) (Figure 2, Table S4). In general, the concentration of total phenolic precursors in the AC increased as the days of treatment increased (Figure 2, Table S4).

Factors such as individual gut microbiota composition, exposure time, and concentration are major determinants of how hydroxycinnamic acids and flavonols are metabolized in the gut [47]. Regarding flavanols, catechin, which is a minor compound in the undigested HPP-apple ingredient (Table S3), was extracted during the entire treatment period in the TC in a range from 2.32 to 7.71 µg/100 g fw of the slurry. The presence of these catechin levels in the TC may be due to the conversion of epicatechin into catechin [47,48].

Quercetin aglycone, an important flavonol for its health-related properties [12], was a minority in the undigested HPP-apple ingredient (0.23 µg/g dw) (Table S3), but significant amounts of this compound were found in the AC and TC during the entire treatment period (14 d) (Table 1). Considering that the GID-CF system was fed each day with 37.5 g of the HPP-apple ingredient, representing an 8.26 µg daily supply of quercetin, and that the apple quercetin bioaccessibility was about 50% [39,40], the daily amount of quercetin that would be reached by the colon simulator could be much less than 4 µg (50% of 8.26 µg). The quercetin concentration found in the AC, TC, and DC slurries during the 14-day treatment period (Table 1) was likely formed by the action of enzymes from the gut microbiota (β-glucosidases, β-glucuronidases, and α-rhamnosidases) that break down quercetin glycosides [49].

Similar results were found for an HPP-onion submitted to a GID-CF process, although the concentration of quercetin in the AC was higher in the onion than the apple due to the higher content of quercetin glycosides in the nondigested onion [38]. Also, a quick deglycosylation of quercetin glycosides present in different fruits, including ‘Golden Delicious’ apples, using static models with human fecal samples to simulate colonic fermentation has been referenced by different authors [28,50].

In general, quercetin deglycosylation depends on the concentration of quercetin derivatives, sugar moieties, inter-individual variation in the microbiota profile, and the food matrix, which could modulate the interaction between the phenolic compound and the gut microbiota [46,51,52].

#### 3.2.2. Evolution of Phenolic Compound Metabolites During Colonic Fermentation

**Figure 3:**
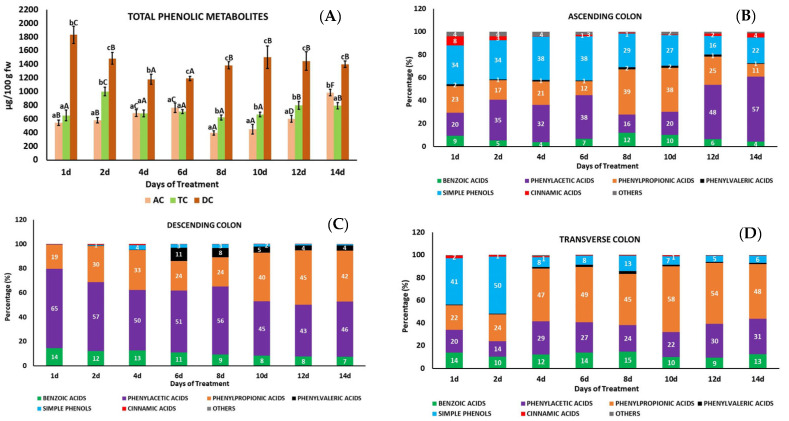
Total phenolic metabolites (**A**) and individual phenolic metabolites grouped by families (**B**–**D**) found in the fermentation slurry of the ascending, transverse, and descending colon (AC, TC and DC). Data are the mean values ± standard deviations of three different determinations (*n* = 3). Different lowercase letters indicate significant differences (*p* < 0.05) between AC, TC, and DC for the same treatment day. Different uppercase letters indicate significant differences (*p* < 0.05) between different treatment days in the same colon regions (AC, TC, and DC). The percentage (%) of each phenolic metabolite family was calculated with respect to the total phenolic metabolites in the same colon region (AC, TC, and DC) and treatment day. Only metabolite families with a percentage ≥ 1% are shown in the bars of the graphs.

A total of 24 phenolic metabolites were identified and quantified using HPLC-ESI-QTOF-MS/MS (Tables S2 and S5), which were monitored in the three colon regions (AC, TC, and DC) during the whole treatment period. The phenolic metabolites identified belonged to different phenolic compound families, mainly derivatives of phenylacetic and phenylpropionic acids and simple phenols (phloroglucinol and catechol). Those in minor concentration included derivatives of benzoic, cinnamic, and phenylvaleric acids and others (quinic acid and dihydroquercetin) (Figure 3B–D, Table S2).

The bioconversion of phenolic compounds by the gut microbiota is highly variable because of their chemical structure, the composition of microbial communities, and the capacity of the microbiota to produce different enzymes involved in numerous reactions (hydrolysis, hydrogenation, α- and β-oxidation, demethoxylation, dihydroxylation, decarboxylation) that result in the formation of a wide range of metabolites [53]. While most of the phenolic precursors were found in the AC (Figure 2), the phenolic metabolites were found in the DC, in significantly higher concentrations, followed by the TC (Figure 3A). These metabolites were mainly derivatives of phenylacetic acid, followed by phenylpropionic and benzoic acids (Figure 3D). The highest concentration of phenolic metabolites found in DC vessel of the GID-CF system employed in this study was consistent with what happens in this part of the large intestine during a human digestion. DC is the final part of the large intestine, where waste moves slowly towards the rectum, allowing for greater compaction of the fecal material before their elimination and in consequence a highest concentration of gut metabolites is accumulated.

At the beginning of the treatment, the highest contributors to the total metabolite content in the AC and TC were simple phenols (mainly phloroglucinol), followed by derivatives of phenylacetic and phenylpropionic acids (Table S5, Figure 3B,C). The simple phenol concentrations in the AC and TC progressively decreased as the treatment days progressed; meanwhile, phenylacetic and phenylpropionic acid derivatives increased in the AC and TC, respectively. At the same time, the concentration of phenylacetic acid derivatives in the DC decreased in favor of phenylpropionic acid derivatives as the treatment days progressed (Figure 3D). 

A brief description of the characteristics of the phenolic compound metabolites organized by families is provided below.

*Simple phenols*: Two compounds were identified, mainly in the AC and TC (Table S5, Figure 3B,C), mostly phloroglucinol and, at a lower concentration, catechol. Phloroglucinol results from the metabolism of quercetin, procyanidins, chalcones, and dihydrochalcones [54,55]. Phloroglucinol can be formed from any flavonoid with a hydroxyl group at the 5 and 7 positions of the A-ring [54], and catechol can be formed by the decarboxylation of protocatechuic acid (3,4-dihydroxybenzoic acid) [56].

*Phenylacetic acid derivatives*: The metabolites in the highest concentration in the three regions of the colon during the 14-day treatment belonged to this family. The main metabolites were 4-hydroxyphenylacetic acid, 4-hydroxy-3-methoxyphenylacetic acid (homovanillic acid), 3-hydroxyphenylacetic acid, 3,4-dihydroxyphenylacetic acid, and phenylacetic acid (Table S5). The metabolite at the highest concentration of all the metabolites identified in the three colon regions was 4-hydroxyphenylacetic acid, although the greatest concentration was in the DC on day 1 (768.05 ± 8.98 µg/100 g fw) and day 14 (418.03 ± 9.10 µg/100 g fw) (Table S5). Homovanillic acid had the highest concentration in the AC (88.85 ± 1.93 µg/100 g fw) and progressively increased up to treatment day 14 (504.37 ± 1.30 µg/100 g fw) (Table S5). The presence of homovanillic acid has been identified in the degradation pathways of quercetin aglycone [49]. Phenylacetic acid derived metabolites have been identified as the main metabolites resulting from the catabolism of quercetin and quercetin glycosides by the intestinal microbiota [49,57]. Also, 3,4-dihydroxyphenylacetic acid has been described in the metabolism of caffeic acid derivatives [58]. 4-hydroxyphenylacetic acid has been reported as a common metabolite in the degradation pathways of hydroxycinnamic acids, such as chlorogenic acid (5-caffeoylquinic acid), and flavonoids, such as quercetin derivatives [57,59]. Both compounds are present at high concentrations in apples.

The phenolic metabolites of this group have various beneficial effects on health. For example, 3,4-dihydroxyphenylacetic acid has antiproliferative, anti-inflammatory, hepatoprotective, and antioxidant characteristics, as assayed in in vitro and in vivo studies [60,61].

*Phenylpropionic acid derivatives*: The main metabolites identified were 3-(3-hydroxyphenyl)-propionic acid, 3-(4-hydroxyphenyl)-propionic acid, 3,4-dihydroxyphenylpropionic acid (dihydrocaffeic acid), and phenylpropionic acid (Table S2). These metabolite compounds are present in different degradation pathways of phenolic compounds [53,57,62].

The metabolite 3-(3-hydroxyphenyl)-propionic acid was identified in the AC, TC, and DC throughout the treatment period and has been described in the chlorogenic acid metabolic degradation pathway [63].

Phenylpropionic acid was detected in the TC but mainly in the DC. This compound is the final metabolite of different phenolic compound degradation pathways, although it has also been detected as a fermentation product in the basal intestinal medium. This metabolite has been identified in the colonic fermentation of several products rich in phenolic compounds, such as wine, grapes, and tea, but also in whites (water and nutrient medium) [64].

Some metabolites in this family have important health-promoting properties, such as 3-(3-hydroxyphenyl)-propionic acid, which is a potent vasodilator, even more so than quercetin [65].

*Benzoic acid derivatives*: The major metabolites identified were 3,5-dihydroxybenzoic acid and 3,4-dihydroxybenzoic acid (protocatechuic acid), followed by salicylic acid, 3-hydroxybenzoic acid, 4-hydroxybenzoic acid, syringic acid, gallic acid, and benzoic acid (Table S2). These metabolites have been identified as the final degradation pathway products of different phenolic compounds due to intestinal microbiota fermentative processes [54,57,66].

*Cinnamic acid derivatives*: Caffeic acid, ferulic acid, and isoferulic acid were found in the colonic fermentation vessels of the HPP-apple ingredient (Table S5). The presence of caffeic acid during the treatment period in the three colon regions could be related to chlorogenic acid (5-caffeoylquinic acid) metabolism, which is the most abundant phenolic compound in apples [1]. Caffeic acid was also described as a phenolic microbial metabolite during the in vitro batch culture fermentation of different fresh apple varieties, albeit its content decreased significantly after 5 h of incubation [28]. Isoferulic acid was found in the three colon regions during the 14-day intake period, but at a lower concentration than caffeic acid. Ferulic and isoferulic acids have been described as products of caffeic acid ester metabolism [67].

*Phenylvaleric acid derivatives*: The metabolites identified were 3-hydroxy-5-(phenyl)-valeric acid, mainly at the end of the treatment period in the DC, and 4-hydroxy-5-(phenyl)-valeric acid, mainly in the AC (Table S5). These two hydrodroxyphenylvaleric acids, together with hydroxyphenyl-γ-valerolactones, have been described as initial or intermediate gut metabolites of flavanols [68]. In the present study, hydroxyphenyl-γ-valerolactones were not detected due to their quick bioconversion to hydroxyphenylvaleric acids by the gut microbiota [48]. Considering the low bioaccessibility of flavanols, since a high percentage reaches the colon unchanged (>70%), it could be that their metabolites, such as phenylvalerolactones and phenylvaleric acids, were mainly responsible for the positive health effects related to the intake of flavanol-rich foods, such as the cardioprotective effect, among others [69].

*Other metabolites*: In this group, the other metabolites included quinic acid and dihydroquercetin (Table S5). The presence of quinic acid, together with caffeic acid, could be the result of chlorogenic acid (5-caffeoylquinic acid) metabolism by the gut microbiota. Dihydroquercetin (or taxifolin) was only detected at the end of the 14-day intake period in the CA and TC groups. This compound, which is the initial metabolite formed from quercetin, is practically undetectable since it is rapidly metabolized to form di- and hydroxyphenylpropionic acids [57,70].

#### 3.2.3. Multivariate Analysis of the HPLC-ESI-QTOF-MS/MS Data

**Figure 4:**
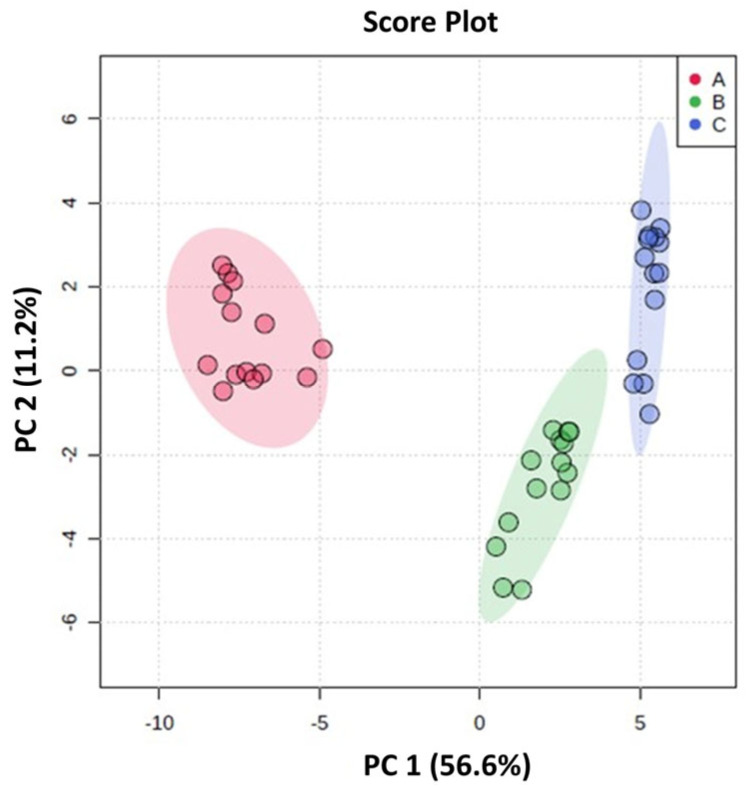
Principal component analysis (PCA) score plot of HPP-apple colonic fermentation products. The parameters for these models are R2 = 0.823 and Q2 = 0.796. A Student’s *t*-test at a 95% level (*p* < 0.05) was used to select the most important variables in each model.

A total of 46 features were identified in the HPP-apple colonic fermentation samples using HPLC-ESI-MS-QTOF-MS in negative ionization mode. Among these, 22 were precursors of phenolic compounds, and 24 were phenolic metabolites (Table S2). The PCA identified a model that clearly separates the components into three different clusters. This indicates that the three colonic regions, i.e., the AC, TC, and DC, have very different compositions of phenolic compounds (precursors) and their corresponding metabolites (Figure 4). The data variability was mainly explained by the first component (PC1) (56.6%). The multivariate model showed good quality in terms of explained variance (R2 = 0.823) and predicted variance (Q2 = 0.796).

### 3.3. Effects of the HPP-Apple Ingredient on the Gut Microbiota

The effect of the reciprocal relationship between phenolic compounds and dietary fiber on the intestinal microbial community is well established [51,71]. Approximately 90–95% of apple phenolic compounds are not absorbed in the small intestine; together with dietary fiber, they reach the large intestine unaltered, where they interact with the intestinal microbiota. The phenolic compounds that reach the colon are mainly catabolized, forming low-molecular-weight bioactive metabolites, as described in the previous section, and those metabolites influence the gut microbiota composition [72,73].

**Figure 5:**
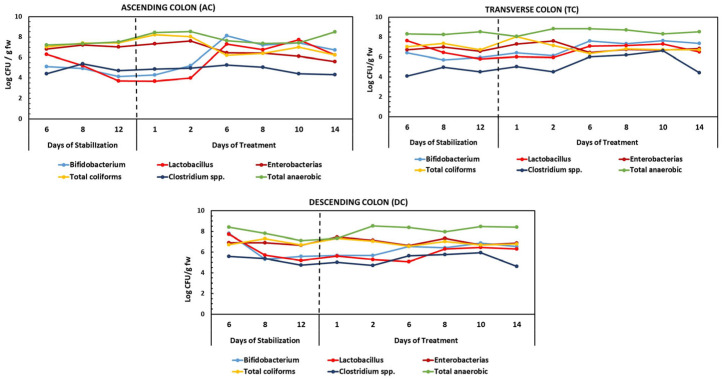
Changes in bacterial populations during the stabilization and treatment periods with the HPP-apple ingredient (37.5 g) in the ascending (AC), transverse (TC), and descending colon (DC). The results are expressed as log CFU/g fw (logarithm of colonic-forming units per gram of fresh weight of slurry).

The influence of the HPP-apple ingredient on the gut microbiota population was evaluated using plate counts; however, more accurate molecular methods exist, such as DNA sequencing [72,73]. Considering the limitations of the plate count method, differences are considered statistically significant when the increase is greater than log (CFU/mL) ≥ 1. The gut microbiota analyzed were *Enterobacteriaceae* and anaerobic bacteria, representing indicators of the total colon microbiota, *Bifidobacterium* spp. and *Lactobacillus* spp., representing beneficial bacteria, and *Clostridium* spp. and total coliforms, representing potentially harmful colon bacteria (Figure 5).

The results showed that the stabilization of the human fecal microbiota in the AC, TC, and DC compartments of the dynamic gut fermentation model was achieved 13 days after incorporating the culture medium (Figure 5). During the treatment period (14 days) with 37.5 g of the HPP-apple ingredient per day, a significant increase in the anaerobic bacteria counts (1 log CFU/mL) was observed in the AC and DC, while it was stable in the TC compared to the stabilization period (basal level). At the same time, the level of *Enterobacteriaceae* decreased by about 1.5 log CFU/g fw of slurry in the AC but remained stable in the TC and DC, after 14 days of treatment compared to the basal level. Also, the level of *Clostridium* spp. decreased slightly in the AC (0.5 log CFU/g fw), but no changes were observed in the TC or DC at the end of the treatment period, with respect to the basal level. The total coliform content was reduced in the AC (1 log CFU/g fw) and remained stable in the TC and DC (Figure 5).

Therefore, the 14-day daily treatment with 37.5 g of HPP-apple seemed to favor a microbiota composition balance by increasing *Bifidobacterium* spp. and *Lactobacillus* spp., which are beneficial microbiota bacteria, and a tendency to decrease *Enterobacteriaceae*, total coliforms, and *Clostridium* spp. levels, which are considered harmful colon bacteria. In fact, the levels of *Bifidobacterium* spp. and *Lactobacillus* spp. increased in the three regions of the colon, with the most significant increment observed in the AC (2.5 log CFU/g fw) for both.

In line with the present results, previous studies have shown that whole-apple in vitro batch-culture fermentation of different varieties (Renetta Canada, Golden Delicious) using human fecal portions significantly changed the bacterial diversity of the human microbiota. Specifically, a significant decrease (*p* < 0.05) in *Bacteroidetes* and a greater increase in *Bifidobacterium* spp. were observed, the latter even higher than that observed after inulin (a known prebiotic) administration [30].

Also, the beneficial effects of isolated phenolic compounds and rich phenolic sources, such as tea, berries, pomegranate, grapes, cocoa, and red wine, among others, on intestinal microbiota were recently reviewed [73]. In general, phenolic compounds (catechin, epicatechin, anthocyanins, caffeic acid, chlorogenic acid, quercetin, rutin, naringenin, etc.) and rich sources of these compounds (berries, tea, cocoa, grapes, pomegranate, etc.) have prebiotic effects as they increase the growth of beneficial bacteria, such as *Lactobacillus* and *Bifidobacterium* spp. and reduce the abundance of harmful bacteria, such as certain *Clostridium* species [66,74,75,76]. Therefore, these results highlight the beneficial effect of the 14-day daily incorporation of the HPP-apple ingredient on the intestinal microbiota.

### 3.4. Effects of the HPP-Apple Ingredient on Short-Chain Fatty Acids (SCFAs)

The major SCFAs analyzed were acetic acid (C2:0), propionic acid (C3:0), and butyric acid (C4:0). Other minor fatty acids analyzed were isobutyric acid (C4:0), valeric acid (C5:0), caproic acid (C6:0), and heptanoic acid (C7:0). The major SCFA content represented more than 95% of the total fatty acids analyzed in the colon fermentation samples, which were studied as indicators of microbiota activity and metabolism. Current evidence suggests that SCFAs can regulate human body metabolism, maintain the intestinal barrier, reduce inflammation, and ameliorate the effects of various diseases, among other effects [77,78].

**Figure 6:**
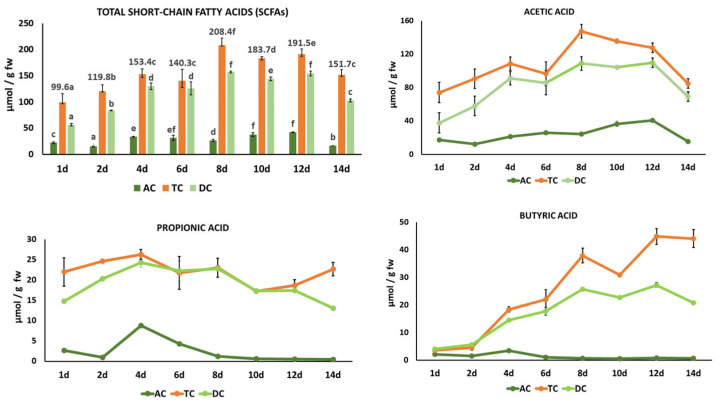
Evolution of total and individual short-chain fatty acids (acetic, propionic, and butyric acids) in the ascending colon (AC), transverse colon (TC), and descending colon (DC) during 14 days of treatment with the HPP-apple ingredient (37.5 g). Different letters indicate significant differences (*p* < 0.05) between different treatment days in the same colon region (AC, TC, and DC). Data are the mean values ± standard deviations (µmol/g fw of slurry) of three different determinations (*n* = 3).

In general, total SCFAs significantly increased (*p* < 0.05) in the three colon regions during the 14-day intake period with the HPP-apple ingredient, albeit their content was higher in the TC than in the AC and DC (Figure 6). The formation of fatty acids is more abundant in the proximal colon (ascending and transverse colon) than in the distal colon (descending colon). In fact, in the present study, the highest concentration was mainly quantified in the TC and gradually decreased from the proximal to the distal colon (DC). These results are in line with others found in the literature. For example, the content of acetic, propionic, and butyric acids analyzed in an in vitro cranberry and grape seed extract fermentation sample represented more than 90% of the total fatty acids studied. In this case, the SCFA content was higher in the DC than in the AC [66].

In the present study, the highest SCFA concentration was observed on treatment day 8 in the TC (Figure 6), where acetic, propionic, and butyric acids were present in a molar ratio of 74:11:18. This proportion was similar to that described by other authors (60:25:15), although proportions can vary depending on factors such as diet, microbiota composition, colon region, and host genotype [78].

The evolution of the individual SCFAs studied is shown in Figure 6. Acetic acid was the major SCFA in the three colon regions throughout the treatment period, with significantly higher levels in the TC than in the AC and DC. The acetic acid peak content in the TC on treatment day 8 (147.45 ± 8.27 µmol/g fw) was 6.4 and 3.8 times higher than propionic acid and butyric acid, respectively, on the same treatment day. Also, the acetic acid content reached a peak content in the DC on treatment day 8 (108.91 ± 1.32 µmol/g fw), and its concentration remained stable between day 8 and day 12. From day 12 to day 14, acetic acid significantly decreased by 68%, 34%, and 37% in the AC, TC, and DC, respectively. Propionic acid showed non-significant differences between the TC and DC from day 4 and day 8. The concentration of propionic acid in the TC was relatively stable between day 4 and day 14, with a medium value of 22.50 µmol/g fw.

The significant increase in butyric acid of 13 times in the TC and 10 times in the DC from treatment day 1 (3.52 ± 0.51 and 2.07 ± 0.03 µmol/g fw, respectively) to treatment day 14 is noteworthy (44.09 ± 3.23 and 20.77 ± 0.24 µmol/g fw, respectively) (Figure 6).

The high butyric acid production is of great interest, as this fatty acid is increasingly recognized as a key regulator of intestinal health, brain function, inflammation, and even cancer prevention [77]. Interestingly, not all apples or their derivatives have this effect. For example, several apple varieties subjected to an in vitro batch fermentation process increased the acetic and propionic acid levels, but only one variety (‘Renetta Canada’) increased the butyric acid level to a much greater extent compared to those observed for inulin and cellulose [28]. Other authors have also observed an increase in butyric acid during colonic fermentation of phenolic-rich compounds, such as tea, coffee, olive leaf, rosemary, and oregano [70,79]. This result could be due to the action of phenolic compounds on butyrate-producing bacteria, particularly those belonging to the phyla *Firmicutes* and *Actinobacteriota*.

In general, the 14-day continuous feeding with the HPP-apple ingredient resulted in a significant increase in the total SCFAs of 2 times in the TC and 3 times in the DC compared with the initial content on treatment day 1. The significant increase of 13 times the initial content of butyric acid in the DC is significant due to its recognized beneficial health effects.

### 3.5. Interaction Patterns Between Phenolic Metabolites, Gut Microbial Groups, and SCFA Production

**Figure 7:**
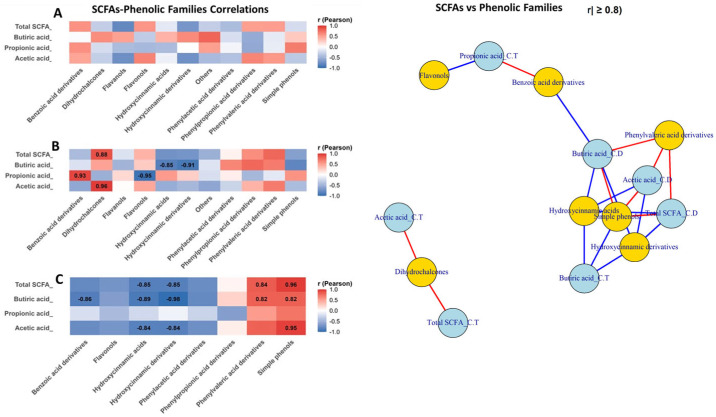
Differential SCFAs–phenolic class interactions along an in vitro colon model: correlation heatmaps and network analysis. This figure integrates Pearson correlation heatmaps and a correlation network to illustrate the relationships between SCFAs–phenolic class interactions along an in vitro colon model: ascending colon (**A**), transverse colon (**B**) and descending colon (**C**). Positive correlations indicate that SCFAs and phenolic classes co-vary in the same direction, whereas negative correlations reflect inverse associations consistent with microbial biotransformation of polyphenols. In the network representation, nodes correspond to SCFA classes (blue) or phenolic families (yellow), and edges depict significant correlations (red = positive; blue = negative), with edge thickness proportional to the correlation strength.

Pearson correlation analysis revealed clear section-dependent associations between phenolic compound classes, SCFAs production, and gut bacterial composition. Figure 7 shows the Pearson’s correlation between SCFAs levels and the different classes of phenolic compounds (PC) in each section of the colon (AC, TC and DC) (Figure 7A–C). In colon ascendant (Figure 7A), correlations were generally weak, whereas transverse colon (Figure 7B), several strong positive associations emerged, particularly the correlations of individual SCFA as propionic acid (*r* = 0.93), acetic acid (*r* = 0.96), total SCFAs (*r* = 0.88) with metabolites belonging to the families of benzoic acid, phenylacetic acid and phenylvaleric acid derivatives and simple phenols, while hydroxycinnamic derivatives showed consistently negative correlations (*r* = −0.91). In colon descendent (Figure 7C), SCFAs displayed strong negative correlations with hydroxycinnamic and phenylacetic acid derivatives (*r* between −0.84 and −0.98), together with strong positive associations with simple phenols (*r* up to 0.95) and phenylvaleric derivatives (*r* = 0.82–0.84).

**Figure 8:**
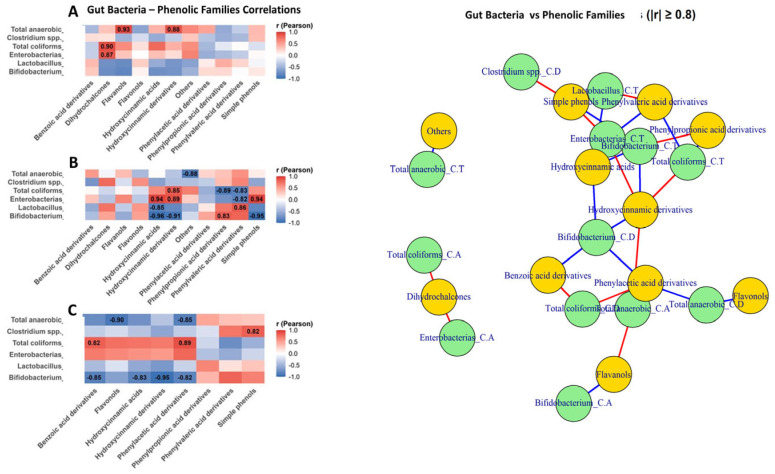
Differential gut bacteria–phenolic class interactions along an in vitro colon model: correlation heatmaps and network analysis. This figure integrates Pearson correlation heatmaps and a correlation network to illustrate the relationships between gut bacterial groups (*Bifidobacterium*, *Lactobacillus*, *Enterobacteriaceae*, *Clostridium* spp., total anaerobes, and total coliforms) and phenolic compound families across the three colonic sections: ascending colon (**A**), transverse colon (**B**) and descending colon (**C**). Positive correlations indicate co-variation between bacterial abundance and phenolic classes, whereas negative correlations represent inverse relationships consistent with microbial utilization or degradation of polyphenols. In the network representation, nodes correspond to bacterial groups (green) and phenolic classes (yellow), and edges denote significant correlations (red = positive; blue = negative), with edge thickness proportional to correlation strength.

Bacterial correlations followed similar patterns (Figure 8). Thus, in ascending colon (Figure 8A), the correlations were weaker and more heterogeneous, with positive associations between *Enterobacteriaceae* and dihydrochalcones (*r* = 0.87) and between total anaerobic and benzoic acid derivatives (r = 0.93). In transverse colon (Figure 8B), *Enterobacteriaceae* showed strong positive correlations with phenylpropionic and phenylvaleric derivatives (*r* = 0.89–0.94), whereas *Bifidobacterium* and *Lactobacillus* showed strong negative correlations with hydroxycinnamic and benzoic acid derivatives (*r* down to −0.96). In descending colon (Figure 8C)., coliforms remained positively associated with simple phenols (*r* = 0.82–0.89). Also, *Bifidobacterium* and *Lactobacillus* showed positive correlations with phehylvaleric derivatives and simple phenols (Figure 8C)

Network analysis (|r| ≥ 0.8) confirmed two main clusters: one linking fermentative bacteria and SCFAs with phenylpropionic, phenylvaleric acid derivatives, and simple phenols (positive edges), and another dominated by hydroxycinnamic and benzoic acid derivatives, connected through negative correlations involving *Bifidobacterium*, *Lactobacillus*, and several SCFAs.

## 4. Conclusions

Feeding a dynamic in vitro gastrointestinal digestion and colonic fermentation simulator (GID-CF) for 14 days with 37.5 g of the HPP-apple ingredient (400 MPa/5 min/35 °C) resulted in an initial accumulation of phenolic compounds precursors in the AC (mainly chlorogenic acid, epicatechin, quercetin-rhamnoside, quercetin-galactoside, and phloridzin). This quickly disappeared and yielded a high content of total phenolic metabolites in the ascending and transverse colon (AC, TC) and in a 3.3 times higher concentration in the descending colon (DC) due to colonic metabolism. The phenolic compound biotransformation yielded phloroglucinol, 4-hydroxyphenylacetic acid, 4-hydroxy-3-methoxyphenylacetic acid, 3-(3-hydroxyphenyl)-propionic acid, and 3-(4-hydroxyphenyl)-propionic acid as the main metabolites of gut microbiota catabolism at different concentrations depending on the colon region. These phenolic metabolites are related to significant benefits for human health.

The three colon regions (AC, TC, and DC) were perfectly differentiated based on their content of phenolic compound precursors and their corresponding metabolites, according to a PCA. An important modulatory effect of the 14-day continuous intake of the HPP-apple ingredient on the gut microbiota was the increase in lactic acid bacteria, including *Bifidobacterium* spp. and *Lactobacillus* spp., both associated with the beneficial microbiota of the colon. This increase, observed in the AC, TC, and DC, reached up to 2.5 log CFU/g fw after 14 days of treatment compared to the baseline period. Added to this beneficial effect was the significant increase in SCFAs, mainly butyric acid, which also has important beneficial effects on human health.

These findings highlight the potential prebiotic-like effect that the HPP-apple ingredient has on the gut microbiota and its transformation into phenolic metabolites with important benefits for human health.

## Figures and Tables

**Table 1:** Quercetin content in three colon regions (AC, TC, and DC) during a dynamic in vitro gastrointestinal digestion and colonic fermentation of the HPP-apple ingredient.

Daily Quercetin Supply (µg)		8.62
Colon Region	Day	Quercetin (µg/100 g fw)
AC ^1^	4 d	27.19 ± 0.84 ^c^
	9 d	5.05 ± 0.28 ^c^
	14 d	3.82 ± 0.37 ^c^
TC ^1^	4 d	6.97 ± 0.44 ^a^
	9 d	1.79 ± 0.02 ^b^
	14 d	2.56 ± 0.17 ^b^
DC ^1^	4 d	23.12 ± 0.09 ^b^
	9 d	1.28 ± 0.14 ^a^
	14 d	0.99 ± 0.09 ^a^

^1^ AC—ascending colon, TC—transverse colon, DC—descending colon. Data are expressed as mean ± standard deviation of two different determinations of two different digestions (n = 4); fw, fresh weight. Different small letters indicate significant differences (*p* < 0.05) between different colon regions (AC, TC, and DC) on the same day.

## Data Availability

The original contributions presented in this study are included in the article and Supplementary Materials. Further inquiries can be directed to the corresponding author.

## Supplementary Materials

The following supporting information can be downloaded at https://www.mdpi.com/article/10.3390/metabo15120775/s1, Table S1: Physicochemical, chemical, and biochemical characteristics of the undigested untreated and HPP-apple; Table S2: HPLC-ESI-QTOF-MS/MS identification of phenolic compound precursors and metabolites in the undigested HPP-apple ingredient and in the fermentation slurry of the digested HPP-apple ingredient subjected to a dynamic in vitro gastrointestinal digestion and colonic fermentation; Table S3: Phenolic compound content (µg/g dw) in the undigested HPP-apple ingredient; Table S4: Phenolic precursor content (µg/100 g fw) in the gut fermentation slurry of the three colon regions (AC, TC, and DC) during the HPP-apple ingredient digestion; Table S5: Phenolic metabolite content (µg/100 g fw) in the gut fermentation slurry of the three colon regions (AC, TC, and DC) during the HPP-apple ingredient fermentation.
