# Supplementary material for: Effect of High-Pressure Processed Apples on Phenolic Metabolites, Short-Chain Fatty Acids, and Human Gut Microbiota Using a Dynamic In Vitro Colonic Fermentation System
Source: Metabolites. 2025 Nov 29;15(12):775. doi: 10.3390/metabo15120775 (PMC12734438; doi:10.3390/metabo15120775)
Supplement: Supplementary file 1 [file metabolites-15-00775-s001.zip › TABLE S1-Physicochemical, chemical, biochemical-161125.pdf]

Table S1. Physicochemical, chemical, and biochemical characteristics of the undigested untreated and HPP-treated apple.

Parameters	Untreated	HPP-treated
**Physicochemical characteristics**		
pH	3.64 ± 0.05[a]	3.72 ± 0.03[a]
Acidity (g malic acid/100 g dw)	0.75 ± 0.03[a]	0.64 ± 0.01[a]
**Chemical characteristics**		
Ascorbic acid (mg/100 g dw)	17.12 ± 0.34[b]	13.53 ± 0.39[a]
Vitamin C (mg/100 g dw)	48.76 ± 0.66[b]	46.70 ± 0.78[a]
Protein (g/100 g dw)	1.65 ± 0.06[b]	0.24 ± 0.02[a]
**Fibre**		
Soluble fiber (mg/100 g dw)	3.14 ± 0.20[a]	3.91 ± 0.45[b]
Insoluble fiber (mg/100 g dw)	11.29 ± 0.25[b]	10.88 ± 0.30[a]
Total fiber (mg/100 g dw)	14.43 ± 0.35[a]	14.79 ± 0.24[a]
Total pectin (mg/100 g dw)	2.60 ± 0.48[a]	2.80 ± 0.30[b]
Esterification degree pectin (%)	37.80 ± 5.90[a]	50.00 ± 3.30[b]
**Sugars**		
Sacarose (g/100 g dw)	6.09 ± 0.58[a]	4.85 ± 0.77[a]
Glucose (g/100 g dw)	10.17 ± 0.62[a]	9.67 ± 1.18[a]
Fructose (g/100 g dw)	23.67 ± 1.88[a]	20.38 ± 2.33[a]
**Organic acids**		
Malic acid (g/100 g dw)	2.13 ± 0.04[a]	1.84 ± 0.02[a]
Citric acid (mg/100 g dw)	33.15 ± 2.25[a]	32.11 ± 2.51[a]
Fumaric acid (mg/100 g dw)	9.45 ± 0.38[a]	9.05 ± 2.33[a]
Acetic acid (mg/100 g dw)	2.32 ± 0.39[a]	2.01 ± 0.24[a]
**Biochemical characteristics**		
Enzymatic activity		
PPO (ΔDO/min/g dw)	0.44 ± 0.02[a]	0.42 ± 0.11[a]
POD (ΔDO/min/g dw)	8.81 ± 0.39[b]	4.84 ± 0.84[a]

Values are the mean ± standard deviation (n=4); dw, dry weight; HPP, High-pressure processing at 400 MPa/5 min/35 °C; PPO, polyphenoloxidase; POD, peroxidase; ΔDO/min, optical density increment per minute. Different letters in the same row indicate significant differences ($p < 0.05$) between untreated and HPP-treated apple.
