# Supplementary material for: Effect of High-Pressure Processed Apples on Phenolic Metabolites, Short-Chain Fatty Acids, and Human Gut Microbiota Using a Dynamic In Vitro Colonic Fermentation System
Source: Metabolites. 2025 Nov 29;15(12):775. doi: 10.3390/metabo15120775 (PMC12734438; doi:10.3390/metabo15120775)
Supplement: Supplementary file 1 [file metabolites-15-00775-s001.zip › TABLE S2-Identification by HPLC-ESI-QTOF-MSMS-161125.pdf]

Table S2. HPLC-ESI-QTOF-MS/MS identification of phenolic compounds precursors and metabolites in the undigested HPP-apple ingredient and in the fermentation slurry of the digested HPP-apple ingredient subjected to a dynamic *in vitro* gastrointestinal digestion and colonic fermentation.

Compounds	Formula	Mass	RT(min)	*m/z* (M-H)	Mayor ESI *m/z* [M-H]- and fragments
**Phenolic compound precursors in the undigested HPP-apple ingredient and in the gut fermentation slurry**					
**Flavonols**					
Q-3-Galactoside	$C_{21}H_{20}O_{12}$	464.0969	15.38	463.0881	463.088; 300.0273; **301.0349**; 255.14; 271.0257; 151.0004
Q-3-Rutinoside	$C_{27}H_{30}O_{16}$	610.1550	15.38	609.1457	609.1471; 553.3327; 463.0928; **301.1196**
Q-3-Glucoside	$C_{21}H_{20}O_{12}$	464.0963	15.70	463.0884	463.0888; 343.0571; **301.0319**; 271.0205; 229.0334
Q-3-Arabinoside	$C_{20}H_{18}O_{11}$	434.0855	16.14	433.0777	433.0795; 415.1971.341.1133; **301.0339**; 271.0221; 151.0075
Q-3-Xyloside	$C_{20}H_{18}O_{11}$	434.0854	16.41	433.0777	433.0782; 416.9801; 346.0838; **301.0303**; 283.0768; 233.4208
Q-3-Rhamnoside	$C_{21}H_{20}O_{11}$	448.1014	17.07	447.0937	447.0932; **301.0324**; 284.0311; 283.1580
Quercetin (Q)	$C_{15}H_{10}O_7$	302.0381	19.97	301.0357	301.0340; 273.0260; 245.0376; **178.9937**; **151.0054**; 121.0313; 107.0123
**Hydroxycinnamic acids**					
Neochlorogenic acid	$C_{16}H_{18}O_9$	354.0951	4.49	353.0878	353.0885; 191.0585; 179.0410; 173.0462; 91.0585
Chlorogenic acid	$C_{16}H_{18}O_9$	353.0881	6.02	353.0877	353.08905; 336.86028; 309.8683; 291.868; **191.0554**; 87.0787
Cryptochlorogenic acid	$C_{16}H_{18}O_9$	354.0949	7.77	353.0878	353.0833; **191.0518**; 179.0340; 173.0437
Coumaric acid	$C_9H_8O_3$	164.0460	10.87	163.0400	163.0388; 145.9291; 129.9739**; 119.0363**; 102.94814; 96.9467
p-Coumaroyl-quinic acid	$C_{16}H_{18}O_8$	338.0997	11.08	337.0923	337.0923; 319.0818; 293.1025; 291.0869; 275.0919; **191.0556**; 173.045; 163.0395; 145.0290; 87.0083
**Flavanols**					
Procyanidin B1	$C_{30}H_{26}O_{12}$	578.1424	4.39	577.1351	577.1360; 424.9118; **289.0700**; 179.0559
Catechin	$C_{15}H_{14}O_6$	290.0794	5.36	289.0717	289.0724; **245.0813**; 270.8308; 191.0536; 167.03521
Procyanidin B2	$C_{30}H_{26}O_{12}$	578.1430	10.19	577.1351	577.3602; 425.0868; **289.0697**; 178.9776

Epicatechin	$C_{15}H_{14}O_6$	290.0787	10.95	289.0717	289.0727; **245.0787**; 203.0781; 179.0435; 137.0248; 125.0236; 109.0306
**Dihydrochalcones**					
3-hydroxyphloretin-2'-*O*-xyloxyl-glucoside	$C_{26}H_{32}O_{15}$	584.1733	15.43	583.1661	583.1667; 301.0356; 300.0334; 271.0182
3-hydroxyphloretin-2'-*O*-glucoside	$C_{21}H_{24}O_{11}$	452.1293	16.49	451.1240	451.1256; 433.1607; 301.0423
Phloretin-2'- xyloglucoside	$C_{26}H_{32}O_{14}$	568.1801	16.99	567.1714	567.1737; 549.2539; 433.0899; **273.0780**; 225.0633; 149.0471; 59.0156
Phloretin-pentoxyl-hexoside	$C_{26}H_{32}O_{14}$	568.1808	17.27	567.1711	567.1720; 537.1320; **273.0790**
Phloridzin	$C_{21}H_{24}O_{10}$	436.1377	18.18	435.1296	435.1311; 288.9734; 259.1113
Phloretin	$C_{15}H_{14}O_5$	274.0847	21.54	273.0768	273.0654; 179.034; **167.0357**; 123.0333; 119.0384; 81.024
**Phenolic compounds metabolites in the gut fermentation slurry**					
**Benzoic acid derivatives**					
3,4-Dihydroxybenzoic acid	$C_7H_6O_4$	154.0243	1.89	153.0193	153.0199; 133.0260; **109.0291**; 91.0226; 81.0286
Salicylic acid (2-hydroxybenzoic acid)	$C_7H_6O_3$	138.0330	2.99	137.0244	137.0266; 129.0563; 119.0363; 112.9852; **93.0347;** 89.0252; 68.9969
4-Hydroxybenzoic acid	$C_7H_6O_3$	138.0322	3.49	137.0257	137.0284; 124.0280; 112.9876; 108.1550; 98.9575; **93.0345**
3,5-Dihydroxybenzoic acid	$C_7H_6O_4$	154.0267	5.26	153.0193	153.0267;137.0336; 131.0709; 109.0295**; 93.00381;** 68.9951
3-Hydroxybenzoic acid	$C_7H_6O_3$	138.0323	5.53	137.0255	137.0251; 123.0451; 121.0295; **93.0351**; 91.0046
Benzoic acid	$C_7H_6O_2$	122.0364	12.51	121.0294	121.0646;102.9488; 89.0262; **77.0457**
**Phenylacetic acid derivatives**					
3,4-dihydroxyphenylacetic acid	$C_8H_8O_4$	168.0425	2.50	167.035	167.0343; **123,0447**; 137.0271; 96.4594; 61.9887
3-Methoxy-4-hydroxyphenylacetic acid (Homovanillic acid)	$C_9H_{10}O_4$	182.0586	2.70	181.0506	181.0584; 167.0358; **137.0305**; 123.0461; 122.03504
4-Hydroxyphenylacetic acid	$C_8H_8O_3$	152.0474	4.66	151.0407	151.0534; 137,0281; 128.0355; 121.0295; 112.9862; **107.0497;** 96.9503
3-Hydroxyphenylacetic acid	$C_8H_8O_3$	152.0478	6.18	151.0406	151.0406; **107.0512**; 112.9861; 108.0542; 119.0363; 65.0368

Phenylacetic acid	$C_8H_8O_2$	136.0527	12.99	135.0451	135.0452; 128.03566; 117.21072; 105.0039; **91.0479**; 65.0357
**Phenylpropionic acid derivatives**					
Dihydrocaffeic acid (3,4-dihydroxyphenylacetic acid)	$C_9H_{10}O_4$	182.0544	4.79	181.0471	181.0517; **137.0257**; 121.0274; 119,0363; 112.9832; 109.0270
3-(3-hydroxypheny)-propionic acid	$C_9H_{10}O_3$	166.0602	9.61	165.0557	165.0417; 147.3718; 137.0349; **121.0909**; 107.4270
3-(4-hydroxypheny)-propionic acid	$C_9H_{10}O_3$	166.0618	11.59	165.0551	165.0462; 148.3569; **121.0439**; 107.0339; 92,8287
Phenylpropionic acid	$C_9H_{10}O_2$	150.0665	18.18	149.0607	149.0689; **105.2957**; 133.4147; 88.1326; 74.0109
**Phenylvaleric acid derivatives**					
3-hydroxy-5-(phenyl)-valeric acid	$C_{11}H_{14}O_3$	194.0917	21.38	193.0867	193.0854; 176.5354; **149.660**; 59.6636
4-hydroxy-5-(phenyl)-valeric acid	$C_{11}H_{14}O_3$	194.0917	23.68	193.0867	193.0759; 175.9572; 152.9061; **148.6544**; 59.0124
**Simple phenols**					
Phloroglucinol	$C_6H_6O_3$	126.0335	0.81	125.0249	125.0214; **108.3153**; 121.3252; 83.0178
Catechol	$C_6H_6O_2$	110.0366	2.70	109.0298	109.0308; **108.0225**; 96.9602; 91.0153**;** 89.0244
**Hydroxycinnamic derivatives**					
Caffeic acid	$C_9H_8O_4$	180.0446	6.31	179.0356	179.0367; 161.0325; **135.04595**;134.0401; 109.0283; 61.9901
Ferulic acid	$C_{10}H_{10}O_4$	194.0584	12.99	193.0514	193.0521; 177.0602; 164.9641; **149.0641**; 134.0384
Isoferulic acid	$C_{10}H_{10}O_4$	194.05700	16.81	193.0506	193.0515; 178.9886; **149.2177**; 96.9549
**Others**					
Quinic acid	$C_7H_{12}O_6$	192.0644	0.55	191.0551	191.0556; **173.0450**; 147.0293; 145.0501; 129.0188; 87.0446
Dihydroquercetin	$C_{15}H_{12}O_7$	304.0557	14.27	303.051	303.0528; 285.0332; 260.9191; **151.9627; 125.0254**; 110.9089
