# Supplementary material for: Effect of High-Pressure Processed Apples on Phenolic Metabolites, Short-Chain Fatty Acids, and Human Gut Microbiota Using a Dynamic In Vitro Colonic Fermentation System
Source: Metabolites. 2025 Nov 29;15(12):775. doi: 10.3390/metabo15120775 (PMC12734438; doi:10.3390/metabo15120775)
Supplement: Supplementary file 1 [file metabolites-15-00775-s001.zip › TABLE S3-Phenolic compound in HPP-apple-161125.pdf]

Table S3: Phenolic compound content (μg/g dw) in the undigested HPP-apple ingredient.

Compounds	HPP-apple ingredient
Phenolic acids	
Neochlorogenic acid	198.53 ± 2.53
Chlorogenic acid	368.29 ± 0.86
Cryptochlorogenic acid	6.99 ± 0.30
p-Coumaric acid	0.54 ± 0.03
p-Coumaroyl quinic acid	111.53 ± 2.41
Protocatechuic acid	0.17 ± 0.01
Salicylic acid	0.62 ± 0.05
Total phenolic acids	686.13 ± 6.16
Flavanols	
Procyanidin B1	10.96 ± 0.32
Catechin	5.87 ± 0.12
Procyanidin B2	126.74 ± 0.51
Epicatechin	234.51 ± 0.03
Procyanidin C1	56.58 ± 0.70
Tetramer of epicatechin	10.85 ± 0.08
Dimer of epicatechin	17.07 ± 0.25
Total flavanols	462.58 ± 2.01
Flavonols	
Q-3-rutinoside	1.92 ± 0.02
Q-3-galactoside	66.08 ± 0.93
Q-3-glucoside	13.02 ± 0.28
Q-3-arabinoside	24.84 ± 0.53
Q-3-xyloside	2.04 ± 0.09
Q-3-rhamnoside	79.74 ± 0.32
Quercetin (Q)	0.23 ± 0.00
Total flavonols	187.81 ± 2.17
Dihydrochalcones	
3-Hydroxyphloretin-2'-xyloglucoside	2.40 ± 0.24
3-Hydroxyphloretin-2'-glucoside	2.18 ± 0.01
Phloretin-2'-xyloglucoside	24.81 ± 0.02
Phloretin-pentoxyl-hexoside	4.34 ± 0.11
Phloridzin	53.91 ± 0.46
Phloretin	1.99 ± 0.01
Total dihydrochalcones	86.93 ± 0.84
Total Phenolic Compounds	1426.15 ± 11.184

Data are expressed as the mean ± standard deviation (n=4);
Q, quercetin; dw, dry weight; HPP, High-pressure processing at 400 MPa/5 min/35 °C.
