# Supplementary material for: Effect of High-Pressure Processed Apples on Phenolic Metabolites, Short-Chain Fatty Acids, and Human Gut Microbiota Using a Dynamic In Vitro Colonic Fermentation System
Source: Metabolites. 2025 Nov 29;15(12):775. doi: 10.3390/metabo15120775 (PMC12734438; doi:10.3390/metabo15120775)
Supplement: Supplementary file 1 [file metabolites-15-00775-s001.zip › TABLE S4-Phenolic precursor content-161125.pdf]

Table S4. Phenolic precursor content (μg/100 g fw) in the gut fermentation slurry of the three colon regions (AC, TC, and DC) during the HPP-apple ingredient digestion.

Compounds	Colon region	1d	2d	4d	6d	8d	10d	12d	14d
**Flavonols**									
Q-3-rutinoside	AC	6.17±0.1^{cd}	6.82±0.48^{cd}	8.12±0.54^{cd}	9.15±0.84^{d}	7.77±0.71^{cd}	7.32±1.41^{bc}	nd	1.51±0.10^{a}
	TC	nd	nd	nd	nd	nd	nd	nd	nd
	DC	nd	nd	nd	nd	nd	nd	nd	nd
Q-3-galactoside	AC	86.76±0.81^{e}	58.39±3.88^{d}	0.48±0.22^{a}	28.66±2.46^{bc}	111.92±16.47^{f}	125.76±16.22^{f}	2.88±0.28^{a}	25.77±3.47^{bc}
	TC	nd	nd	nd	nd	nd	2.43±0.09^{c}	0.60±0.03^{a}	1.19±0.09^{b}
	DC	1.78±0.18	nd	nd	nd	nd	nd	nd	nd
Q-3-glucoside	AC	3.30±0.21^{d}	7.78±0.35^{f}	nd	1.30±0.03^{ab}	1.67±0.55^{b}	2.22±0.39^{c}	nd	nd
	TC	nd	nd	nd	nd	nd	nd	nd	nd
	DC	nd	nd	nd	nd	nd	nd	nd	nd
Q-3-arabinoside	AC	10.82±0.26^{cde}	13.50±0.76^{ef}	16.51±0.85^{g}	10.30±0.33^{bcd}	7.90±0.88^{bc}	8.98±1.04^{bcd}	nd	4.07±0.52^{a}
	TC	nd	nd	nd	nd	nd	nd	nd	nd
	DC	nd	nd	nd	nd	nd	nd	nd	nd
Q-3-xiloside	AC	2.11±0.15^{bcd}	2.53±0.10^{cde}	3.23±0.20^{de}	2.78±0.19^{de}	2.57±0.42^{cde}	2.87±0.39^{de}	0.66±0.19^{a}	1.82±0.25^{bc}
	TC	nd	0.71±0.49^{a}	1.50±0.20^{c}	0.98±0.02^{ab}	0.92±0.01^{ab}	1.00±0.10^{ab}	nd	nd
	DC	nd	nd	nd	nd	nd	nd	nd	nd
Q-3-rhamnoside	AC	58.82±2.44^{ab}	71.68±4.51^{bcd}	85.58±0.85^{de}	71.27±2.53^{bcd}	76.92±14.60^{bcde}	93.51±13.10^{e}	51.75±4.62^{a}	92.89±10.40^{e}
	TC	1.07±0.31	nd	nd	nd	nd	nd	nd	nd
	DC	0.65±0.18	nd	nd	nd	nd	nd	nd	nd
**Phenolic acids**									
Neochlorogenic A.	AC	40.11±40^{e}	28.93±0.44^{e}	10.08±1.08^{d}	5.42±0.29^{abcd}	4.66±0.49^{abc}	8.48±2.07^{cd}	2.23±0.16^{a}	8.06±0.10^{bcd}
	TC	2.93±0.14^{ab}	4.37±0.23^{c}	2.85±0.37^{a}	nd	nd	nd	nd	nd
	DC	nd	nd	nd	n.d	nd	nd	nd	nd
Chlorogenic A.	AC	196.60±11.30^{h}	141.48±5.71^{fg}	71.88±1.05^{bcd}	76.05±6.62^{cd}	117.48±10.90^{ef}	211.62±39.50^{h}	7.24±1.10^{a}	154.37±5.63^{g}
	TC	10.25±1.96^{a}	14.67±2.31^{b}	14.35±0.86^{b}	nd	nd	nd	nd	nd
	DC	nd	nd	nd	nd	nd	nd	nd	nd
Criptochlorogenic A	AC	111.28±6.88^{j}	89.41±0.87^{i}	28.31±0.89^{g}	19.95±1.55^{def}	14.77±1.76^{cd}	26.75±6.04^{h}	4.04±0.42^{a}	24.99±0.94^{efg}
	TC	14.51±2.90^{b}	21.95±1.57^{c}	10.56±0.70^{a}	nd	nd	nd	nd	nd
	DC	nd	nd	nd	nd	nd	nd	nd	nd
p-Coumaric A.	AC	2.08±0.03^{a}	2.21±0.18^{a}	2.51±0.02^{a}	1.89±0.12^{a}	3.2±0.46^{bc}	4.33±0.68^{d}	6.43±0.48^{e}	2.34±0.01^{a}
	TC	2.20±0.28^{abc}	4.71±0.26^{f}	3.84±0.27^{e}	3.16±0.14^{d}	2.41±0.08^{bc}	3.91±0.20^{e}	1.87±0.22^{ab}	4.04±0.44^{a}
	DC	2.46±0.11^{d}	1.02±0.07^{c}	5.26±0.38^{e}	0.61±0.04^{a}	nd	nd	nd	nd
p-Coumaroil quinic A.	AC	70.29±2.36^{cd}	72.77±4.94^{cd}	104.59±2.18^{e}	79.73±5.75^{d}	117.17±13^{ef}	125.99±14.50^{f}	32.22±4.14^{ef}	113.38±9.1^{ef}
	TC	4.15±0.16^{d}	10.71±1.53^{e}	15.44±0.98^{g}	3.72±0.16^{cd}	1.01±0.03^{a}	1.10±0.06^{a}	1.33±0.09^{ab}	1.95±0.14^{abc}
	DC	nd	nd	nd	nd	nd	nd	nd	nd

**Flavanols**									
Procyanidin B1	AC	6.51±0.26^{c}	8.47±2.03^{cd}	7.61±0.27^{cd}	1.03±0.07^{a}	1.99±0.15^{ab}	2.88±0.13^{ab}	1.42±0.41^{a}	6.64±2.50^{c}
	TC	nd	nd	nd	nd	nd	nd	nd	nd
	DC	nd	nd	nd	nd	nd	nd	nd	nd
Catechin	AC	13.45±0.58^{g}	13.43±0.61^{g}	6.70±0.24^{d}	2.19±0.23^{a}	3.07±0.23^{ab}	5.24±0.97^{bc}	3.74±0.23^{bc}	8.43±0.01^{e}
	TC	2.32±0.27^{a}	7.71±0.18^{g}	3.90±0.39^{de}	3.95±0.25^{de}	4.24±0.09^{e}	3.05±0.10^{bc}	4.24±0.17^{e}	2.64±0.22^{ab}
	DC	nd	nd	nd	nd	nd	nd	nd	nd
Procyanidin B2	AC	56.83±0.28^{e}	67.71±0.14^{fg}	64.54±0.93^{ef}	5.41±0.38^{a}	15.63±2.81^{ab}	26.53±0.85^{cd}	12.53±1.9^{ab}	74.34±3.08^{g}
	TC	nd	19.70±0.42^{d}	20.57±3.31^{d}	0	0	9.44±0.99^{b}	8.72±0.35^{b}	nd
	DC	nd	nd	nd	nd	nd	nd	nd	nd
Epicatechin	AC	58.99±4.81^{i}	53.94±2.78^{i}	28.72±1.33^{g}	2.54±0.37^{a}	8.56±0.60^{abc}	22.89±3.75^{fg}	10.76±1.33^{bcd}	55.80±0.76^{i}
	TC	1.61±0.02^{ab}	3.56±0.04^{d}	5.08±0.53^{e}	1.39±0.13^{a}	1.49±0.90^{ab}	1.75±0.06^{ab}	nd	nd
	DC	nd	nd	nd	nd	nd	nd	nd	nd
**Dyhidrochalcones**									
3-Hydroxyphloretin-2'-xyloglucoside	AC	5.70±0.19^{cd}	5.25±0.47^{abcd}	5.88±0.26^{cd}	nd	5.44±0.42^{bcd}	5.16±0.15^{abc}	nd	5.58±0.57^{ac}d
	TC	nd	nd	nd	nd	nd	nd	nd	nd
	DC	nd	nd	nd	0	nd	nd	0	nd
3-Hydroxyphloretin-2'-glucoside	AC	5.13±0.45^{abc}	4.62±0.17^{ab}	nd	5.31±0.05^{abc}	5.41±0.39^{abc}	5.69±0.17^{bc}	4.54±0.31^{ab}	5.02±0.54^{ab}
	TC	nd	nd	nd	nd	nd	nd	nd	nd
	DC	nd	nd	0	nd	nd	nd	nd	nd
Phloretin-2'-xyloglucoside	AC	8.16±0.68^{c}	7.46±0.51^{bc}	7.11±0.64^{bc}	6.63±0.38^{b}	7.43±0.19^{bc}	7.16±0.47^{bc}	nd	4.88±0.47^{a}
	TC	nd	nd	nd	nd	5.28±0.28^{c}	4.96±0.26^{b}	nd	nd
	DC	nd	nd	nd	nd	0	0	0	nd
Phloretin-pentoxyl-hexoxide	AC	47.06±2.34^{i}	45.73±1.84^{i}	39.11±2.38^{h}	11.61±0.79^{bc}	19.4±1.27^{ef}	13.38±1.54^{c}	4.82±0.61^{a}	5.02±0.54^{ab}
	TC	nd	0	0	0	0	0	0	0
	DC	nd	0	0	0	0	0	0	0
Phloridzin	AC	23.40±0.55^{c}	47.33±1.53^{f}	4.80±0.53^{ab}	24.92±2.72^{c}	34.37±5.83^{d}	41.85±7.73^{ef}	6.16±0.38^{ab}	12.18±0.78^{b}
	TC	nd	nd	nd	nd	nd	nd	nd	nd
	DC	nd	nd	nd	nd	nd	nd	nd	nd
Phloretin	AC	5.06±0.42^{a}	nd	5.02±0.43^{a}	5.09±0.32^{a}	4.89±0.32^{a}	4.85±0.15^{a}	4.56±0.32^{a}	4.57±0.48^{a}
	TC	nd	nd	nd	nd	nd	nd	nd	nd
	DC	nd	nd	nd	nd	nd	nd	nd	nd

Data are expressed as the mean ± standard deviation (n = 3). Lowercase letters mean statistically significant differences ($p < 0.05$) between different days for the same compound; Q, quercetin; A, acid; AC, ascending colon; TC, transverse colon; DC, descending colon; fw, fresh weight, d, day; nd, not detected.
