# Supplementary material for: Effect of High-Pressure Processed Apples on Phenolic Metabolites, Short-Chain Fatty Acids, and Human Gut Microbiota Using a Dynamic In Vitro Colonic Fermentation System
Source: Metabolites. 2025 Nov 29;15(12):775. doi: 10.3390/metabo15120775 (PMC12734438; doi:10.3390/metabo15120775)
Supplement: Supplementary file 1 [file metabolites-15-00775-s001.zip › TABLE S5.Phenolic metabolites content-131125.pdf]

Table S5: Phenolic metabolite content (µg/100 g fw) in the gut fermentation slurry of the three colon regions (AC, TC, and DC) during the HPP-apple ingredient fermentation.

Compounds	Colon regions	1d	2d	4d	6d	8d	10d	12d	14d
**Benzoic acids**									
3,5-Dihydroxybenzoic acid	AC	24.37±1.49^{e}	9.69±0.45^{b}	5.82±0.20^{a}	16.52±1.1^{d}	8.13±0.55^{b}	9.98±2.34^{bc}	11.21±0.90^{c}	15.58± 0.71^{d}
	TC	23.14±5.47^{f}	16.29±0.95^{e}	9.12±0.83^{c}	8.16±0.22^{c}	6.38±0.14^{b}	4.28±0.15^{a}	6.32±0.34^{b}	11.71±0.70^{d}
	DC	100.84±12.2^{f}	48.54±3.01^{e}	22.66±0.35^{d}	13.49±0.73^{c}	10.82±0.63^{b}	8.58±0.43^{a}	8.21±0.60^{a}	9.37±0.49^{a}
3.4-Dihydroxybenzoic acid	AC	7.98±1.45^{b}	2.60±0.01^{a}	6.84±1.02^{b}	15.74±3.54^{c}	17.84±1.33^{c}	16.12±2.41^{c}	14.05±0.28^{c}	3.42±0.48^{a}
	TC	8.33±0.80^{f}	3.63±0.30^{e}	2.05±0.17^{d}	2.17±0.75^{d}	1.57±0.11^{b}	1.92±0.06^{c}	1.37±0.33^{b}	0.42±0.00^{a}
	DC	38.38±9.79^{e}	22.47±1.95^{d}	9.00±0.76^{c}	4.53±0.09^{b}	2.49±0.18^{a}	3.18±0.90^{a}	2.75±0.57^{a}	3.51±1.17^{a}
4-Hydroxybenzoic acid	AC	5.85±0.56^{b}	6.35±0.23^{b}	7.56±0.15^{c}	8.33±0.39^{d}	3.85±0.05^{a}	6.22±1.25^{b}	6.49±0.39^{b}	6.00±0.51^{b}
	TC	12.58±1.90^{a}	16.63±1.85^{c}	13.87±0.88^{ab}	20.09±0.39^{e}	18.57±0.81^{d}	12.91±0.75^{a}	14.21±0.98^{b}	16.45±1.22^{c}
	DC	19.97±2.59^{b}	13.95±1.97^{a}	20.72±1.35^{b}	12.68±0.16^{a}	20.96±1.77^{b}	24.99±1.85^{b}	19.40±0.67^{b}	13.45±0.58^{a}
3-Hydroxybenzoic acid	AC	nd	nd	nd	nd	nd	nd	nd	nd
	TC	5.78±1.04^{c}	9.28±0.20^{d}	6.29±0.40^{c}	8.73±0.33^{d}	5.82±0.16^{c}	2.54±0.22^{a}	2.89±0.36^{a}	4.49±0.30^{b}
	DC	9.07±1.21^{c}	9.22±1.26^{c}	9.02±0.64^{c}	8.89±0.24^{c}	6.81±1.24^{b}	4.02± 0.30^{b}	3.32±0.08^{a}	3.24±0.05^{a}
Salicylic acid	AC	13.08±1.32^{d}	12.86±0.40^{d}	5.73±0.18^{a}	10.24±1.26^{c}	17.23±1.48^{e}	13.45±3.10^{d}	6.42±0.46^{b}	17.74±2.38^{e}
	TC	2.09±0.20^{a}	3.59±0.94^{bc}	3.02±0.25^{bc}	2.98±0.33^{b}	3.14±0.42^{bc}	2.77±0.19^{b}	2.72±0.37^{b}	2.81±0.19^{bc}
	DC	8.41±1.80^{e}	5.80±1.16^{d}	5.54±0.59^{d}	4.19±0.55^{c}	3.05±0.33^{ab}	3.08±0.05^{a}	2.79±0.39^{a}	3.25±0.00^{b}
Benzoic acid	AC	nd	nd	nd	nd	nd	nd	nd	nd
	TC	nd	nd	nd	nd	nd	nd	nd	nd
	DC	38.96±3.16^{a}	51.76±6.91^{bc}	48.00±1.33^{b}	55.49±1.89^{cd}	56.18±2.25^{cd}	42.41±5.08^{b}	46.90±0.90^{b}	63.95±7.63^{d}
**Phenylacetic acids**									
3,4-Dihydroxyphenyl-acetic acid	AC	nd	nd	nd	nd	nd	nd	nd	nd
	TC	15.22±3.27^{a}	50.63±11.4^{d}	69.80±7.88^{e}	51.56±3.68^{d}	37.72±3.07^{b}	33.21±2.31^{b}	38.20±2.32^{b}	41.24±1.76^{c}
	DC	39.92±7.41^{b}	20.15±3.83^{a}	17.56±2.76^{a}	nd	nd	nd	nd	nd
4-Hydroxy-3-methoxyphenyl-acetic acid (Homovanilic acid)	AC	88.85±1.93^{c}	187.57±9.02^{d}	204.96±7.90^{e}	265.53±22.40^{f}	51.43±3.94^{a}	67.15±12.80^{b}	262.68±21.20^{f}	504.37±1.30^{g}
	TC	21.54±1.75^{c}	22.71±1.67^{c}	45.09±2.16^{e}	27.11±0.72^{d}	4.58±0.27^{a}	16.24±0.69^{b}	44.32±1.62^{e}	68.76±2.34^{f}
	DC	80.60±14.10^{g}	18.75±1.33^{e}	14.08±0.83^{d}	5.89±0.88^{c}	2.76±0.47^{b}	1.95±0.08^{a}	5.80±0.65^{c}	21.06±1.73^{f}
4-Hydroxyphenyl-acetic acid	AC	21.24±0.31^{c}	18.49±1.35^{b}	17.61±0.58^{b}	25.59±1.48^{d}	12.60±1.55^{a}	23.41±4.71^{cd}	24.22±1.34^{cd}	54.36±2.50^{e}
	TC	34.80±2.25^{b}	41.83±1.00^{d}	61.78±3.79^{f}	41.12±1.92^{d}	45.47±1.83^{c}	35.98±1.39^{c}	57.74±1.77^{e}	2.91±2.32^{a}
	DC	768.05±8.98^{f}	610.51±0.79^{e}	392.94±16.00^{a}	397.21±5.32^{a}	519.40±14.60^{d}	443.86±49.80^{c}	404.86±7.34^{b}	418.03±9.10^{b}
3-Hydroxyphenyl-acetic acid	AC	nd	nd	nd	nd	nd	nd	nd	nd

	TC	4.42±0.91^{a}	4.68±0.71^{a}	5.22±0.17^{a}	34.48±0.74^{c}	33.16±1.09^{c}	26.56±1.03^{b}	47.21±3.12^{d}	57.26±4.95^{e}
	DC	51.99±1.28	21.97±2.85^{b}	16.57±1.15^{a}	36.21±0.89^{c}	42.86±1.34^{d}	38.48±5.78^{c}	48.96±3-36^{e}	52.78±2.14^{f}
Phenylacetic acid	AC	nd	nd	nd	nd	nd	nd	nd	nd
	TC	9.99±0.19^{c}	nd	nd	nd	nd	7.43±0.24^{b}	6.67±0.13^{b}	1.46±0.33^{a}
	DC	261.21±27.3^{c}	171.37±15.3^{c}	144.38±6.82^{a}	168.02±7.59^{c}	211.86±6.18	189.22±18.50^{c}	157.07±7.25^{b}	146.29±0.90^{a}
**Phenylpropionic acids**									
3,4-Dihydroxyphenylpropionic acid	AC	nd	nd	nd	nd	nd	nd	54.62±6.04^{b}	28.01±1.10^{q}
	TC	15.22±3.27^{b}	50.63±11.40^{de}	69.80±7.88^{e}	51.56±3.68^{d}	37.72±3.07^{c}	33.21±2.31^{c}	38.20±2.32^{c}	1.24±1.76^{a}
	DC	17.03±2.50^{d}	49.09±0.62	23.86±3.28^{e}	6.11±0.81^{a}	7.85±0.36^{b}	17.18±2.42^{d}	24.07±1.98^{e}	10.81±0.77^{c}
3-(3-Hydroxhphenyl)-propionic acid	AC	126.54±14.5^{c}	98.49±7.27^{b}	141.74±4.60^{cd}	93.60±6.09^{b}	155.35±7.55^{d}	172.58±35.10^{e}	94.34±0.24^{b}	79.84±7.02^{a}
	TC	15.26±3.28^{a}	50.63±11.40^{de}	69.80±7.89^{e}	51.56±3.68^{d}	37.72±3.07^{b}	33.21±2.31^{b}	38.20±3.32^{bc}	41.24±1.76^{c}
	DC	257.52±14.1^{e}	88.37±12.60^{c}	88.64±5.98^{c}	86.47±1.04^{c}	41.95±1.38^{a}	59.76±6.64^{b}	100.90±1.55^{d}	148.74±5.50
3-(4-Hydroxhphenyl)-propionic acid	AC	nd	nd	nd	nd	nd	nd	nd	nd
	TC	28.63±2.27^{a}	43.63±1.46^{b}	135.56±8.68^{c}	190.88±6.49^{de}	223.54±22.80^{e}	172.35±6.98^{d}	205.16±21.80^{e}	202.41±5.41^{e}
	DC	69.57±7.82^{a}	253.23±26.8^{c}	244.76±20.00^{c}	137.93±2.24^{b}	230.09±11.20^{c}	487.24±55.50^{e}	489.60±98.90^{e}	399.75±18.20^{d}
Phenylpropionic acid	AC	nd	nd	nd	nd	nd	nd	nd	nd
	TC	nd	nd	nd	4.52±0.05^{c}	5.67±0.09^{d}	3.25±0.16^{b}	1.69±0.04^{q}	3.44±0.30^{b}
	DC	1.88±0.25^{a}	1.96±0.24^{a}	4.53±0.12^{b}	48.92±1.78^{e}	42.00±1.36^{d}	19.67±2.54^{c}	20.27±0.81^{c}	20.95±1.38^{c}
**Phenylvaleric acids**									
3-Hydroxy-5-(phenyl)-valeric acid	AC	nd	nd	nd	nd	nd	nd	nd	nd
	TC	nd	nd	nd	7.85±0.21^{c}	10.09±0.18^{d}	5.36±0.24^{b}	2.13±0.10^{a}	5.98±0.25^{b}
	DC	nd	nd	nd	125.30±2.57^{d}	100.89±4.20^{c}	70.26±6.90^{b}	56.64±2.40^{a}	53.23±1.00^{a}
4-Hydroxy-5-(phenyl)-valeric acid	AC	8.31±1.01^{e}	3.99±0.25^{a}	9.25±0.91^{e}	5.48±0.57^{b}	6.89±0.32^{c}	8.49±0.13^{d}	8.66±0.97^{d}	6.57±0.02^{c}
	TC	2.74±0.19^{a}	5.36±0.29^{c}	8.95±1.15^{e}	6.19±0.17^{d}	4.90±0.76^{c}	4.50±1.4^{c}	4.85±0.35^{c}	3.01±0.49^{b}
	DC	1.47±0.09^{a}	2.52±0.49^{b}	3.41±0.28^{bc}	3.87±0.76^{bc}	3.32±0.04^{c}	5.05±0.69^{d}	3.37±0.06^{c}	5.74±0.64^{d}
**Simple Phenols**									
Phloroglucinol	AC	177.69±10.8^{b}	189.67±16.45^{b}	251.58±30.00^{c}	287.09±29.50^{c}	115.18±14.20^{a}	116.05±6.15^{a}	99.05±13.90^{a}	215.39±30.60^{abc}
	TC	264.29±18.0^{b}	490.84±2.19^{c}	35.68±4.57^{a}	33.54±4.89^{a}	34.01±3.05^{a}	34.01±3.05^{a}	31.83±2.41^{a}	31.33±2.41^{a}
	DC	nd	10.61±2.07^{b}	24.61±4.09^{c}	26.71±2.08^{c}	38.44±1.78^{d}	26.61±3.39^{c}	14.01±1.93^{b}	4.05±0.55^{a}
Catechol	AC	6.73±0.24^{c}	8.17±0.42^{dc}	7.11±0.86^{c}	7.48±0.25^{c}	0.95±0.11^{a}	3.90±0.87^{b}	nd	4.03±0.81^{b}
	TC	3.92±0.73^{a}	10.55±1.44^{bc}	22.12±1.57^{d}	22.52±0.92^{d}	34.32±1.27^{e}	14.17±1.10^{c}	11.51±0.77^{b}	18.73±1.40^{d}

	DC	3.83±0.61^{b}	3.10±0.51^{b}	18.98±1.41	5.89±0.10^{cd}	7.39±1.18^{d}	5.37±0.78^{cd}	1.88±0.42^{a}	5.06±0.77^{c}
**Cinnamic acids**									
Caffeic acid	AC	39.65±3.08^{e}	19.15±1.94^{d}	1.51±0.66^{a}	6.65±0.97^{b}	nd	nd	10.48±0.74^{c}	36.36±1.48^{e}
	TC	13.45±0.93^{f}	11.97±0.97^{e}	5.16±0.50^{d}	0.57±0.14^{a}	1.47±0.14	2.75±0.15^{c}	0.41±0.04^{a}	1.08±0.28^{b}
	DC	3.80±0.19^{a}	5.84±0.21^{b}	9.80±0.94^{c}	nd	nd	nd	nd	nd
ferulic acid	AC	nd	nd	nd	0.94±0.14^{bc}	1.99±0.11^{d}	1.22±0.34^{c}	0.87±0.24^{b}	0.64±0.03^{a}
	TC	nd	nd	nd	nd	nd	nd	nd	nd
	DC	nd	nd	nd	nd	nd	nd	nd	nd
Isoferulic acid	AC	2.58±0.83^{d}	0.85±0.24^{bc}	1.06±0.17^{c}	0.99±0.35^{c}	0.77±0.00^{b}	0.98±0.11^{c}	0.65±0.03^{a}	0.98±0.06^{c}
	TC	1.82±0.25^{bc}	2.21±0.38^{bc}	1.31±0.39^{b}	1.99±0.40^{bc}	1.84±0.16^{bc}	2.87±0.98^{c}	1.60±0.41^{b}	0.72±0.09^{a}
	DC	3.96±1.01^{d}	2.32±0.18^{c}	1.47±0.27^{c}	2.73±1.33^{abcd}	1.11±0.16^{c}	0.77±0.13^{b}	0.60±0.08^{a}	1.33±0.33^{c}
**Others**									
Quinic acid	AC	22.06±1.87^{c}	23.18±5.25^{c}	27.02±2.7^{c}	21.01±3.94^{c}	1.87±0.05^{a}	9.39±0.66^{b}	8.77±1.00^{b}	10.38±1.24^{b}
	TC	2.85±0.19^{f}	0.82±0.06^{c}	6.75±0.25^{g}	0.68±0.03^{b}	0.46±0.02^{a}	0.57±0.13^{a}	0.94±0.08^{d}	1.34±0.04^{e}
	DC	nd	nd	nd	nd	nd	nd	nd	nd
Dihydroquercetin	AC	nd	nd	nd	nd	1.18±1.12^{ab}	1.31±0.06^{ab}	0.95±0.07^{a}	1.57±0.29^{b}
	TC	nd	nd	nd	nd	nd	0.96±0.06^{b}	0.81±0.12^{a}	0.81±0.06^{a}
	DC	nd	nd	nd	nd	nd	nd	nd	nd

[1] Data are expressed as the mean ± standard deviation (n=3); Lowercase letters mean statistically significant differences ($p < 0.05$) between different days for the same compound; AC, ascending colon; TC, transverse colon; DC, descending colon; fw, fresh weight; d, day; nd, not detected.
